# Induction of Cryptic Antifungal Pulicatin Derivatives from *Pantoea Agglomerans* by Microbial Co-Culture

**DOI:** 10.3390/biom10020268

**Published:** 2020-02-10

**Authors:** Bathini Thissera, Hani A. Alhadrami, Marwa H. A. Hassan, Hossam M. Hassan, Fathy A. Behery, Majed Bawazeer, Mohammed Yaseen, Lassaad Belbahri, Mostafa E. Rateb

**Affiliations:** 1 School of Computing, Engineering & Physical Sciences, University of the West of Scotland, Paisley PA1 2BE, UK; bathini.thissera@uws.ac.uk (B.T.); majed.bawazeer@uws.ac.uk (M.B.); Mohammed.Yaseen@uws.ac.uk (M.Y.); 2 Department of Medical Laboratory Technology, Faculty of Applied Medical Sciences, King Abdulaziz University, Jeddah 21589, Saudi Arabia; 3 King Fahd Medical Research Centre, King Abdulaziz University, Jeddah 21589, Saudi Arabia; 4 Department of Pharmacognosy, Faculty of Pharmacy, Beni-Suef University, Beni-Suef 62514, Egypt; mh_elsefy@yahoo.com (M.H.A.H.); abuh20050@yahoo.com (H.M.H.); 5 Department of Pharmacognosy, Faculty of Pharmacy, Mansoura University, Mansoura 35516, Egypt; fathy.behery@riyadh.edu.sa; 6 Department of Pharmacy, College of Pharmacy, Riyadh Elm University, Riyadh 11681, Saudi Arabia; 7 Laboratory of Soil Biology, University of Neuchatel, 2000 Neuchatel, Switzerland; lassaad.belbahri@unine.ch

**Keywords:** *Pantoea*, *Penicillium*, pulicatin, antifungal, co-culture

## Abstract

Microbial co-culture or mixed fermentation proved to be an efficient strategy to expand chemical diversity by the induction of cryptic biosynthetic pathways, and in many cases led to the production of new antimicrobial agents. In the current study, we report a rare example of the induction of silent/cryptic bacterial biosynthetic pathway by the co-culture of Durum wheat plant roots-associated bacterium *Pantoea aggolomerans* and date palm leaves-derived fungus *Penicillium citrinum*. The initial co-culture indicated a clear fungal growth inhibition which was confirmed by the promising antifungal activity of the co-culture total extract against Pc. LC-HRMS chemical profiling demonstrated a huge suppression in the production of secondary metabolites (SMs) of axenic cultures of both species with the emergence of new metabolites which were dereplicated as a series of siderophores. Large-scale co-culture fermentation led to the isolation of two new pulicatin derivatives together with six known metabolites which were characterised using HRESIMS and NMR analyses. During the in vitro antimicrobial evaluation of the isolated compounds, pulicatin H (**2**) exhibited the strongest antifungal activity against Pc, followed by aeruginaldehyde (**1**) and pulicatin F (**4**), hence explaining the initial growth suppression of Pc in the co-culture environment.

## 1. Introduction

Although natural products have contributed a large number of novel scaffolds with interesting therapeutic values [1], the re-isolation of known compounds has been increasing in recent decades. This is one of the major challenges that pharmaceutical and agricultural research faces in the discovery of novel bioactive compounds [2], emphasising the need for more efficient approaches. Among the various approaches to address this issue, the focus on screening microbes residing in less assessable and extreme environments such as hyper-arid deserts, permafrost soils, deep-sea sediments, highly acidic and hypersaline habitats, is most highlighted [3]. Competition between different microorganisms for food and space is considered to be one of the major ecological forces that induces silent biogenetic gene clusters which triggers the accumulation of cryptic secondary metabolites (SMs) that are not traced in their axenic cultures [4]. In co-cultivation approaches, the activation of cryptic biosynthetic pathways to produce antimicrobial agents are basically caused by creating competition between the species, which are grown in the same environment, predominently for nutrition and space [4]. Although this approach is currently limited due to the fear of reproducibility, one of the advantages of co-culture is that prior knowledge of the signalling mechanism is not required, although direct contact of the two species is vital [5].

It is well-known from our own previous investigations as well as other studies that usually the fungal metabolite pattern is affected in fungal–bacterial mixed fermentations and no clear induction of bacterial cryptic metabolites is observed [6]. Recently, different studies have demonstrated that co-cultivation is a remarkably successful approach for the discovery of new fungal natural products. Our group has demonstrated the efficiency of the co-culture approach when investigating the hyper-arid desert bacterial isolate *Streptomyces bullii* which led to the induction of diverse fungal metabolites that were not traced in the axenic culture [7]. Induction of the cryptic metabolites aspvanicin A and its epimer aspvanicin B was reported from endophytic fungus *Aspergillus versicolor* KU258497 through its co-culture with the bacterium *Bacillus subtilis* 168 trpC2 [8]. Four new naphthoquinone dimers, fusatricinones A–D, and a new lateropyrone derivative, dihydrolateropyrone, were induced in the co-cultivation of the endophytic fungus *Fusarium tricinctum* with *Streptomyces lividans* [9]. It is clear that no induction of cryptic bacterial metabolites was observed through all reported mixed fermentation literature. The very first case reporting the dual induction of cryptic bacterial and fungal metabolites was reported by our group when co-cultivating the marine-derived fungal isolate *Aspergillus fumigatus* MR2012 with the hyper-arid desert bacterial isolate *Streptomyces leeuwenhoekii.* Strain C34 has induced the fungal cryptic metabolites luteoride D and pserotin G together with the bacterial cryptic metabolites chaxapeptin and pentalenic acid [5].

In the current study, we have experienced another rare example of the influence of microbial co-cultures on the induction of cryptic bacterial biosynthetic pathways. Initial co-culture screening between many bacterial and fungal species led us to prioritise two endophytic strains—Durum wheat plant-derived bacterium *Pantoea agglomerans* (Pa) and date palm root-derived *Penicillium citrinum* (Pc)—due to their clear interaction in the solid media growth plate. Dereplication of the co-culture extract of these two strains using LC-HRMS clearly indicated a few new hits that were not traced in the axenic cultures. Following large-scale co-cultivation followed by solvent extraction, different chromatographic purification steps led to the isolation of two new bacterial siderophores belonging to the pulicatin class of compounds, together with two known pulicatin derivatives. Additionally, another four known metabolites belonging to different chemical classes were also isolated. The structures of all the isolated metabolites were then confirmed by HRMS and NMR analysis. Interestingly, the antifungal screening of these isolated metabolites potentially explained the initial growth inhibition of the fungal isolate Pc in the co-culture environment with the bacterium Pa.

## 2. Materials and Methods

### 2.1. General Experimental Procedures

Structure characterization of all compounds was based on ^1^H-NMR, ^13^C-NMR, COSY, HSQC, and HMBC data which were obtained using Bruker Avance III 600 MHz spectrometer. HPLC separations were carried out using a Sunfire^TM^ reversed-phase (C18, 5 µm, 10 × 250 mm, serial No 226130200125) and Agilent, 1100 series gradient pumps and monitored using a DAD G13158B (DE03010630) detector. HRESIMS data were obtained using a Thermo LTQ Orbitrap coupled to an HPLC system (PDA detector, PDA autosampler, and pump). The following conditions were used: capillary voltage of 45 V, capillary temperature of 260 °C, auxiliary gas flow rate of 10–20 arbitrary units, sheath gas flow rate of 40–50 arbitrary units, spray voltage of 4.5 kV, and mass range of 100–2000 amu (maximal resolution of 60,000). Optical rotations were recorded using a Perkin-Elmer 343 polarimeter. UV spectra were obtained using a Perkin-Elmer Lambda2 UV/vis spectrometer.

### 2.2. Bacterial/Fungal Isolation and Mixed Fermentation

Strains Pa and Pc were received from Laboratory of Soil Biology, University of Neuchatel, Switzerland after their isolation and taxonomic identification as previously described [10,11]. Both strains were revived from the glycerol stocks in agar plates with ISP2 medium and incubated for 3 days at 28 °C. Two seed cultures of Pa and Pc were prepared in 1 L Erlenmeyer-flasks supplemented with 250 mL ISP2 medium and left shaking in the rotary shaker at 28 °C for 2 and 4 days, respectively.

Twelve Erlenmeyer-flasks (1 L) were prefilled with a production medium of modified ISP2 (M1, 250 mL) [10] and autoclaved. Every three flasks were inoculated with 10mL of either Pa, Pc, or both strain (co-culture) seed cultures under sterile conditions. The remaining three flasks were used as medium control. All twelve flasks were fermented in the rotary shaker at 180 rpm at 28 °C for eight days. On day eight, 5 g of HP20 resins was added to all flasks, left shaking for 6 h, centrifuged, and the cell mass with the resin were extracted with MeOH to produce the total extracts for the axenic cultures, co-culture and the medium blank. Each extract was defatted by shaking with hexane, and evaporated to dryness under reduced pressure. All microbial extracts and control were prepared for LC-HRMS in triplicates as described before [10]. LC-HRMS data were compared and dereplicated using Xcalibur 3.0 software.

For large scale co-culture fermentation, 40 Erlenmeyer-flasks (1 L) were prefilled with a production medium of modified ISP2 (M1, 250 mL) and autoclaved. All flasks were inoculated with 5 mL of the bacterial and fungal seed culture under sterile conditions and all flasks were fermented in the rotary shaker at 180 rpm at 28 °C for eight days. On the last fermentation day, 5 g of HP20 resins was added to all flasks, left shaking for 6 h, centrifuged and the cell mass with the resin were combined and extracted with MeOH to produce the total co-culture extracts.

### 2.3. Analysis of Raw Mass Data Using MZmine 2.3.7

After acquiring LC-HRMS data for all extracts, the slicing of raw mass data files into two datasets as positive and negative based on ionization mode was performed by using MassConvert tool from ProteoWizard. Only positive mode files were considered for further processing as the negative mode was not high resolution. Sliced data files of positive mode, which included samples and sample blanks of axenic and co-culture extracts, were imported and processed using MZmine 2.3.7 following the steps and predefined parameters explained in [12]. The sequence of steps involved peak detection (mass detection and chromatogram builder), chromatogram deconvolution, deisotoping, filtering, alignment, gap filling, adduct and complex ion search and, finally, formula prediction. CSV file was produced as the final output from the MZmine was further cleaned-up manually by removing adducts and peaks presents in both samples and their medium blanks, as explained in [10,12]. During the elimination of unreliable formulae, the nitrogen rule was also considered. Further cleaning up was carried out by cross-checking the significant intense peaks with Xcalibur 3.0 using raw mass data files. Unidentified peaks by MZmine were also evaluated for reliable formulae and intensity before elimination.

### 2.4. Dereplication Process to Identify Known and New Hits

Alignment of the CSV data into ascending order with the peak retention times was followed by searching databases such as Antibase 2012, SciFinder, Dictionary of Natural product 27.0, DEREP-NP 2013 and REAXYS online databases using predicted formula. This was followed by a thorough search of the fragmentation patterns of the precursor ions on Xcalibur 3.0 with the aid of s fragmentation tool available with ChemDraw professional 16.0 (PerkinElmer Informatics, Cambridge, UK) to make lists of the best possible hits from the two axenic and co-culture total extracts (Tables S1–S3).

### 2.5. Isolation of Compounds

After analysing the LC-HRMS profile of the total extract produced by small-scale mixed fermentation Pa-Pc, large-scale mixed fermentation was performed and the total MeOH extract was obtained as mentioned earlier. After defatting by shaking with hexane, subsequent liquid–liquid fractionation produced two fractions; DCM and EtOAc. As the two fractions DCM and EtOAc were found to have similar HPLC profiles, they were combined, and further purification was achieved using semi-preparative HPLC. HPLC purification of the combined DCM/EtOAc fractions used a semi-preparative column (sunfire^TM^, prep C18, 5 µm, 10 × 250 mm), eluted with a gradient system of 0%–80% CH_3_CN in H_2_O over 35 min, followed by 80%–100% CH_3_CN for 15 min at a flow rate of 1.5 mL/min to yield **1** (t_R_13.2 min, 10 mg), **2** (t_R_17.1 min, 9 mg), **3** (t_R_22.5 min, 2.5 mg), **4** (t_R_16.2 min, 6 mg), **5** (t_R_20.7 min, 7 mg), **6** (t_R_24.9, 4 mg) and **7** (t_R_6.8, 3.5 mg), and **8** (t_R_ 30.3, 4 mg).

Pulicatin H (**2**): White solid; ${\left[\alpha \right]}_{D}^{25}$ + 2.35 (c 0.01, CH_3_OH); UV (MeOH) λ_max_ (log ε) 214 (4.20), 248 (2.92), 294 (3.70), 321 (3.45) nm; ^1^H NMR and ^13^C NMR data, see Table 1; HR-ESI-M (M + H)^+^ at *m/z* 264.0695 (Calc. 264.0689, C_13_H_14_NO_3_S).

Pulicatin I (**3**): yellowish white solid; UV (MeOH) λ_max_ (log ε) 214 (4.25), 249 (2.84), 296 (4.10), 326 (3.15), 344 (2.90) nm; ^1^H NMR and ^13^C NMR data, see Table 1; HR-ESI-M [M + H]^+^ at *m/z* 246.0586 (Calc. 246.0583, C_13_H_12_NO_2_S).

### 2.6. Determination of Absolute Stereochemistry of Compound ***2*** by Modified Mosher’s Method

The reaction was performed according to a modified Mosher ester procedure described by Ohtani et al., 1991 [13]. The pure compound **2** (0.5 mg each) was dissolved in 250 μL of pyridine-*d*_5_ and transferred into clean NMR tubes under N_2_. About 0.5 mg of (+*R*)-α-methoxy-α-(trifluromethyl) phenylacetic acid ((+*R*)-MTPA), was measured, dissolved in 250 μL of pyridine-*d*_5_ under N_2_ and immediately transferred to the NMR tube under a constant N_2_ flow. The NMR tube were shaken carefully and thoroughly to complete the reaction. Preparation and reaction with (-*S*)-MTPA: the pure compound **2** (0.5 mg in 250 μL of pyridine-*d*_5_) was transferred to the NMR tube and the reaction was carried out with (-S)-MTPA, similarly to that described above. The reaction was monitored by ^1^H NMR and COSY after 5-6 h. A pure compound dissolved in Pyridine-*d*_5_ was used as the reference sample to investigate the chemical shift changes.

### 2.7. Antimicrobial Screening (Antibacterial and Antifungal Activities)

#### 2.7.1. Preparation of Samples and Microorganisms Used

The bacterial endophyte *Pantoea agglomerans* isolate Pa and the fungal endophyte *Penicillium citrinum* isolate TDPEF34 (Pc) were obtained from University of Neuchâtel, Switzerland. Additionally, *Candida albicans* NCPF3255 and *Aspergillus niger* IMI51433 were obtained from University of the West of Scotland, Paisley, Scotland, UK. 

All pure compounds isolated were prepared in 5% DMSO by dissolving the freeze-dried compounds into 1 mL of solvent to make stock solutions of 0.5 mM of each. A 5 mg/mL stock solution of total extract was also prepared from co-culture fermentation by dissolving 5 mg of freeze-dried material in 1 mL of 5% DMSO solution. Amphoteracin B (Sigma Aldrich, Gillingham, UK) and Amoxicillin (Sigma Aldrich, UK) were used as positive controls in the antifungal and antibacterial screenings, respectively. The stock solutions (0.5 mM) were prepared in the same manner as the other pure compounds described above. The concentration range for both positive controls were optimized to accommodate the MIC range for the test series. Hence, 1–500 µM for Pc and Pa, and 1–50 µM for *A. niger* and *C. albicans* dilution series, were maintained within the 96-well plates.

#### 2.7.2. Calculation of Minimum Inhibitory Concentration (MIC) Using Microdilution Method

The antimicrobial activity of the total extract, pure compounds (**1**–**8**), including the positive controls, was evaluated by determining the minimum inhibitory concentrations (MIC). The broth microdilution method in sterile 96 well plates (Thermo Scientific Ltd., Loughborough, UK) was performed following the CLSI guidelines [14] and EUCAST guidelines [15] with slight modifications. Briefly, the bacterial (Pa) and fungal (Pc, *A. niger*, *C. albicans*) culture plates were prepared in nutrient agar by streaking under sterile conditions following 24 and 48 h incubation, respectively. The bacterial colony suspension was prepared in Mueller Hinton Broth (MHB), while the medium for fungal colony suspension was RMPI 1640 (Sigma Aldrich, UK) supplemented with 2% glucose. Turbidity of the suspensions were adjusted to 0.5 Mcfarland by 1:100 dilution with respective sterile media (MHB and RMPI 1640). Each 96-well plate included *treatment* (MHB/ RMPI 1640, sample and inoculum), *sterility* control (MHB/ RMPI 1640) only, and *growth* control (MHB/RMPI 1640 and inoculum) in triplicates. The final volume of each well was fixed to 200 µL. To the first three treatment wells, 50 µL of the sample from the stock solution prepared in 5% DMSO was added, and two-fold dilutions were made by serial dilution within the 96-well plate. Each well, except the sterility control and the sample blank (MHB/RMPI 1640+sample), was inoculated with 50 µL of diluted microbial suspension, resulting in the final well inocula of ~5 × 10^5^ cfu/mL. To control the colour difference in the samples, optical density was measured under spectrophotometer (at 570 nm) at t = 0 and after 24 h of incubation at 37 °C. The antibacterial and antifungal effect of the extract and the pure compounds were assessed by calculating the percentage growth of the bacterium/fungi in the treatment wells.

These were in comparison to the growth control, where the growth in the control well was assigned 100% growth (Abs _treatment well_/Abs _control well_ × 100). The growth curves were utilised only to select samples that showed more than 50% growth suppression from the plot, and these were then tested further to pinpoint the accurate MIC value [16]. The analysis of selected compounds was performed by ascertaining the lowest concentration of the well with no visible growth from the selected concentration range. Further iteration was then performed using a second dilution series between the lowest, with no visible growth, and the highest, with visible growth, to obtain an accurate MIC of selected compounds.

## 3. Results

**Figure 1:**
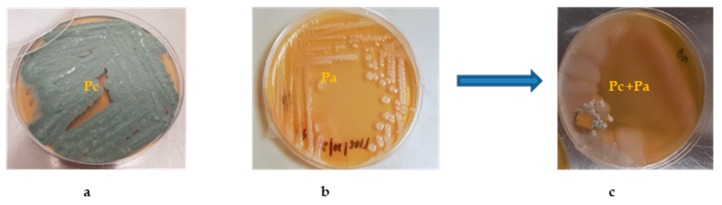
(**a**) Axenic culture of Pc. (**b**) Axenic culture of Pa. (**c**) Co-culture of Pc and Pa, showing a growth inhibition of Pc by Pa.

**Figure 2:**
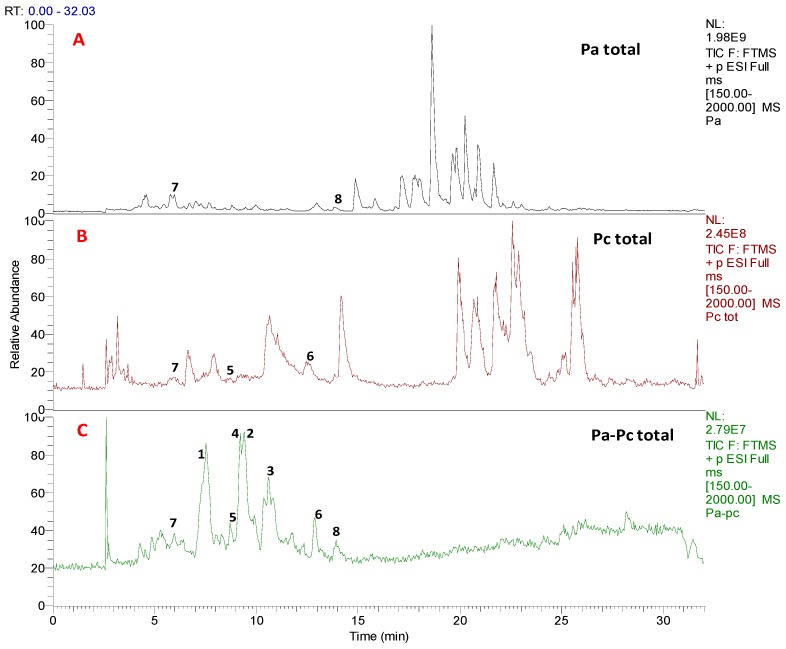
LC-MS profiles of (**A**) axenic Pa; (**B**) axenic Pc; (**C**) Pa-Pc co-culture indicating the isolated compounds **1**–**8**.

During our screening for the discovery of new antibacterial or antifungal natural products, we have used a co-cultivation approach on solid agar plates to access the initial selection of strains used for this purpose. Out of over 20 co-cultivation of different endophytes belonging to the genera *Penicillium* (11 isolates), *Aspergillus* (8 isolates), and *Geotrichum* (1 isolate) recovered from healthy and diseased date palm trees on ISP2 agar solid medium, we have observed that Durum wheat plant-derived bacterium *Pantoea agglomerans* (Pa) invaded all over the culture plate and inhibited the growth of date palm-derived *Penicillium citrinum* (Pc) (Figure 1). This observation encouraged us to initially perform a small-scale mixed fermentation in order to explain the influences on the production of SMs by the interaction of these two species. Additionally, the total extract of the mixed fermentation has shown a clear inhibitory effect against Pc but not Pa, explaining the growth suppression of the fungal species. The initial investigation based on the LC-HRMS analysis revealed a unique chemical profile under mixed fermentation (Figure 2). Dereplication of HRMS data evidenced molecular formulae for new and known series of compounds (Table S1). 

### 3.1. Chemical Profiling and Dereplication of Pa, Pc and Pa-Pc

**Figure 3:**
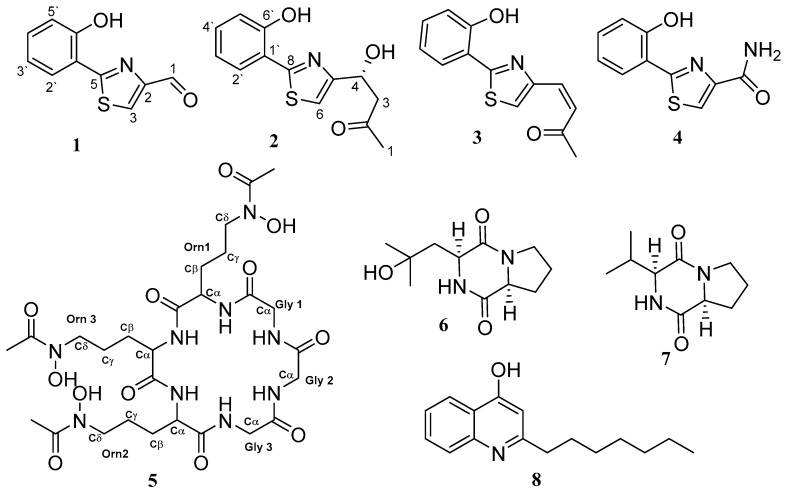
Compounds isolated from the microbial co-culture of Pa and Pc.

Small scale fermentation of Pa, Pc, and Pa-Pc was performed using a modified ISP2 medium (M1) [10]. LC-HRESIMS raw data (Figure 2) files of axenic and co-culture total extracts were processed using MZmine 2.37 to produce a CSV file with possible formulae for peaks detected, along with their *m/z* and retention times. After a thorough clean-up process, as described in [12], the dataset was dereplicated manually, as explained in the experimental section, and the most likely chemical structures were identified by searching the accurate masses and the molecular formulae in the natural product databases. It was interesting to see that the chemical profiles of axenic cultures and co-culture appeared quite distinct (Table S1). Considering the distinctive chemical profile and significant antifungal activity of the co-culture’s total extract, large-scale fermentation was conducted, followed by extraction and multiple steps of chromatographic purification, which afforded the isolation and characterization of eight SMs, some of which were detected in the axenic cultures, and four pulicatin derivatives were induced as bacterial metabolites and were not traced in the LCMC traces of the axenic culture (Figure 3).

### 3.2. Structure Characterisation of the Isolated Compounds

Compound **1** was obtained as a bright yellow solid. The quasimolecular ion peak (M + H)^+^ at *m/z* 206.9937 in the HRESIMS assigned its molecular formula as C_10_H_7_NO_2_S. The ^1^H and ^13^C-NMR data (Table 1) showed resonances for an ortho-disubstituted benzene ring. The signals for aromatic protons were observed at (*δ*_H_ 8.19 (1H, dd (6.1, 1.8) H-2′], (δ_H_ 7.07 (1H, dd (7.5, 1.4) H-3′), δ_H_ 7.37 (1H, t (7.54) H-4′) and (δ_H_ 6.99 (1H, t (7.54) H-5′), and their respective carbons, C-2′ (*δ*_C_127.9), C-3′ (δ_C_ 116.9), C-4′ (δ_C_132.1), and C-5′ (δ_C_ 120.0), were assigned by the correlations in the HSQC spectrum. The possibility of a thiazole ring being one of the substituents to the benzene ring was identified during the dereplication process followed by the literature data survey for the aeruginaldehyde that was the most probable hit [17].

**Figure 4:**
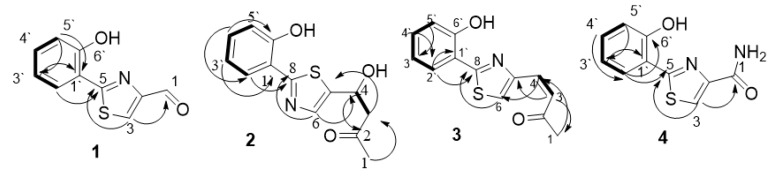
Key COSY (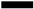) and HMBC (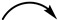) correlation of compounds **1**–**4**.

The structure was further confirmed following a thorough analysis of 2D NMR data. All the protons and carbons were assigned based on HSQC, COSY and HMBC correlations (Figure 4). The resonance of olefinic sp^2^ hybridized methine proton singlet (*δ*_H_ 8.72 (1H, s, H-3) has shown no correlations in the COSY spectrum, implying its attachment to either hetero atoms or quaternary carbons to complete the remaining two sigma bonds. Thus, an olefinic bond was established at C-3 (*δ*_C_131.9).

The HMBC correlation (Figure 4) of H-3 to the low-field quaternary carbon C-5 (*δ*_C_ 164.3) confirmed the presence of the thiazole ring moiety, as reported for aeruginaldehyde. In the ^1^H-NMR, the second low-field aldehyde singlet resonated at *δ*_H_ 10.02 (1H, s, H-1] and was assigned to its aldehyde carbon resonated at a higher chemical shift C-1 (*δ*_C_ 185.1). The HMBC correlations from H-1 to C-2 (*δ*_C_ 153.5) and C-3 (Figure 4) established the attachment of the carbaldehyde moiety to the thiazole ring at C-2. Finally, the attachment of the thiazole ring to the phenyl group was exhibited through C-1′–C-5 bonding through the HMBC correlations of H-2′ to C-5 and C-1′ and H-5′ to C-1′ (Figure 4). The final structure was unambiguously explained the reported compound aeruginaldehyde [17]. Aeruginaldehyde was introduced by Ye et al. as a structure revision of N-mercapto-4-formyl carbostyril isolated from *Pseudomonas fluorescences* as an antibiotic against plant pathogens [17,18].

Compound **2** was obtained as a white solid. HRESIMS data demonstrated its quasimolecular ion peak (M + H)^+^ at *m/z* 264.0695, implying the molecular formula C_13_H_13_NO_3_S. Dereplication with the molecular formula using Antibase, Scifinder and REAXYS databases indicated no hits and potentially new metabolite. The structure characterization was based on in-depth 1D and 2D NMR analysis. In the ^1^H-NMR, spectral resonances for an *O*-disubstituted benzene ring were clearly exhibited. Aromatic methine protons (*δ*_H_ 8.00 (1H, dd (7.88, 1.7), H-2′), (δ_H_ 6.91 (1H, t (6.9), H-3′), (δ_H_ 7.26 (1H, t (7.6), H-4′) and (δ_H_ 7.02 (1H, d (8.7), H-5′) (SI) (Table 1) were assigned to their corresponding carbons and resonated at C-2′ (*δ*_C_ 127.1), C-3′ (δ_C_ 119.8), C-4′ (δ_C_ 131.2), and C-5′ (δ_C_ 117.3) by their H-C correlations in the HSQC spectrum. The correlations in the COSY spectra H-2′ through H-5′ confirmed ABCD aromatic spin system (Figure 4). The aromatic quaternary carbon resonated at C-6′ (δ_C_ 155.4) and explained a phenyl moiety, as demonstrated in compound **1**. The low-field olefinic singlet δ_H 7.48_ (1H, s, H-6] in the ^1^H NMR, and its HMBC correlation to the quaternary carbon C-8 (*δ*_C_ 164.8), supported a thiazole ring as in compound **1**, and this was further supported by the comparable ^13^C chemical resonances with 1 and the number of S and N in the molecular formula. Further analysis of the HMBC correlations established the thiazole ring and its attachment to the benzene ring through C-1′-C-8 (Figure 4). One of the main differences between the proton spectra of compounds **1** and **2** was the absence of the low-field aldehyde proton singlet, but instead there were two resonances for a pair of diastereotopic protons (*δ*_H_ 2.84 (1H, dd (15.3, 8.2) H-3a) and (*δ*_H_2.91 (1H, dd (15.7, 4.62), H-3b), which were assigned as low-field sp^3^ methylene C-3 (*δ*_C_ 51.2), and another signal representing a hydroxylated sp^3^ methine proton (*δ*_H_5.17(1H, q, H-4)). The singlet (*δ*_H_2.16 (1H, s, H-1) in the ^1^H-NMR represented a methyl group, and its high chemical shift explained its presence in an electron withdrawing environment. In the ^13^C-NMR, a signal for a ketone carbon C-2 (*δ*_C_206.5) was noticed. In the HMBC spectrum, the correlations from H-6 to C-4, H-4 to C-2 and C-6 and H-1 to both C-2 and C-3 (Figure 4) and the correlations in the COSY spectrum (Figure 4) established the substructure of 2-hydroxypentanone. The strong HMBC correlation of H-4 to C-6 and H-3 to C-5 confirmed its attachment to the thiazole ring at C-5. Thereby, the final planar structure was assigned. Furthermore, to determine the absolute configuration of the chiral centre bearing a hydroxyl group (secondary alcohol) at C–4, the modified Mosher ester reaction was carried out. The chemical shift difference between (*S*)–MTPA and its (*R*)–MTPA ester, assigned the chiral centre at C–4 to have *S*– configuration (Table 2). The structure was identified as a new pulicatin derivative [19] and assigned as pulicatin H in the pulicatin series.

Compound **3** was obtained as a yellowish white solid. HRESIMS demonstrated its molecular ion peak (M + H)^+^ at 246.0586, assigned as the molecular formula C_13_H_11_O_2_NS. The ^1^H and ^13^C NMR spectral comparison between compounds **2** and **3** indicated a great similarity in their aromatic region. Molecular masses and formulae explained 18 mass unit difference and the formation of compound 3 by removing H_2_O molecule from compound **2**. This was further demonstrated by the appearance of two low-field olefinic protons (*δ*_H_ 7.64 (1H, d (16.9) H-3) and (*δ*_H_ 6.94 (1H, d (17.0) H-4) and their coupling constants, which depicted a *trans* double bond. It was obvious that the hydroxyl moiety 4-OH and the diastereotopic H-3 of compound **2** were replaced by this *trans* double bond in compound **3**. The position of the double bond was further confirmed by the HMBC correlations of 3-H to C-1 and C-5 (Figure 4). The final structure was assigned as dehydroxy pulicatin H. Searching Antibase, Scifinder and REAXYS databases using this structure and the molecular formula indicated this to be a new compound and it was named pulicatin I in the series.

Compound **4** was isolated as a light yellow solid. The molecular formula C_10_H_8_O_2_N_2_S) was assigned by its quasimolecular ion peak (M + H)^+^ at *m/z* 221.0385 in the HRESIMS. The ^1^H and ^13^C NMR spectra showed a clear aromatic region representing a disubstituted benzene ring, which was superimposable with the aromatic regions of compounds **1**–**3** (Table 1), and unambiguously demonstrated an *O*-substituted phenol group. Upon searching natural product and synthetic databases, the only hit matching with the molecular formula and this substructure was pulicatin F, a synthetic pulicatin analogue [19,20], which showed identical ^1^H and ^13^C NMR spectra. The only distinction in the carbon and proton NMR data between compounds **1** and **4** was the replacement of aldehyde carbon C-1, and its proton H-1 of compound **1**, by an amide moiety C-1 (*δ*_C_ 163.3) in compound **4**. All the other 2D correlations (Figure 4) were in full agreement with pulicatin F, and this was its first report as a natural product.

Compound **5** was isolated as a white solid. The molecular formula was deduced as C_27_H_45_N_9_O_12_ by the molecular ion peak (M + H)^+^ appearing at *m/z* 688.3251 in the HRMS. Upon searching for hits matching with the molecular formula, the only hit found in the Antibase explained most of the ^1^H-NMR and 2D NMR spectra, and the reported literature were supportive of the finalization of the structure as desferrichrome. The first report of this compound was in 1952 by Neilands [21] as a pigment, isolated from the rust fungus *Ustilago sphaerogena* as its chelated form with iron. Desferrichrome is a hexapeptide derived from ornithine and is a fungal siderophore. During LC-HRMS dereplication, the formula correspondent to it was identified from the axenic culture of Pc, which further confirmed its origin in Pc and its upregulation by co-culture.

The HRMS data suggested the molecular ion peaks (M + H)^+^ at *m/z* 227.1403 and 197.1285, assigning the molecular formulae as C_11_H_18_N_2_O_3_ and C_10_H_16_N_2_O_2_ for compounds **6** and **7**, respectively. Dereplication using the molecular formulae, the ^1^H and ^13^C NMR spectral data, and optical rotation measurements confirmed that they are the known cyclic dipeptides cyclo(l-Pro-4-hydroxyl-l-Leu) [22] and cyclo(l-pro-l-Val) [23], respectively.

Compound **8** was obtained as a light brown solid. The molecular formula C_16_H_21_NO was demonstrated by the quasimolecular ion peak at (M + H)^+^ *m/z* 244.1713 in the HRMS. The molecular formula and the accurate mass were previously reported from *Pantoea agglomerans* during our metabolomics studies under OSMAC conditions [10]. After careful investigation of all 1D and 2D NMR data, it was unambiguously identified as 2-heptyl-4-hydroxy-quinolone (HHQ) which was first reported in 1998 as a metabolite of a marine sponge-derived *Pseudomonas* species [24].

### 3.3. Antimicrobial Screening

**Figure 5:**
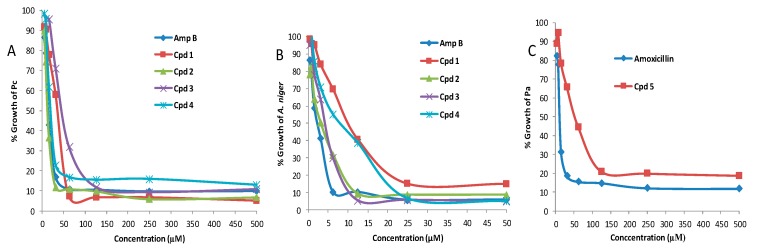
(**A**)**.** Antifungal activities of compounds **1**–**4** against Pc (**B**)**.** Antifungal activities of compounds **1**–**4** against *A. niger* (**C**). antibacterial activity of compound **5** against Pa (X axis represents the concentrations of the sample in µM, Y axis represents the % growth of the microbes (strains Pa, Pc, *A. niger*) in the presence of the sample against the test concentrations).

The co-cultivation total extract and all eight compounds isolated from its large-scale mixed fermentation were assessed for their antifungal activity against Pc, *A. niger*, *C. albicans,* and their antibacterial activity against Pa, to rationalize the incident of fungal growth suppression by Pa. Interestingly, the series of sulfur-containing pulicatin derivatives were found to have good antifungal activity in comparison to positive control amphotericin B when screened against Pc and *A. niger* (Table 3, Figure 5A,B). Compound **2** showed the highest MIC, at 25 and 8.4 μM against Pc and *A. niger*, respectively, followed by compound **1,** with 43 and 40.2 μM against Pc and *A. niger*, respectively. Although compound **3** exhibited good effects, with an MIC of 12.8 μM against *A. niger*, it showed a weak activity against Pc, with an MIC of 127 μM. Compound **4** was the third most active against both Pc and *A. niger,* with MIC values of 53 and 22.6 μM, respectively. The potent antifungal agent Amphoteracin B [20] was used as the positive control and showed MIC values at 16.7 and 5.7 μM against Pc and *A. niger*, respectively. Its antifungal effect was, comparatively, two-, three- and four-fold stronger than compound **2**, **1** and **4**, respectively, against Pc. However, all these bioactive compounds (**1**–**4**) have shown no activity against *C. albicans* up to 50 µM. Additionally, compounds **5**, **6**, **7** and **8** did not show any antifungal effects against Pc, *A. niger* and *C. albicans* within the test concentration series. Interestingly, the co-culture total extract exhibited a promising antifungal activity (MIC-14.9 μg/mL). This could be postulated by possible synergistic behaviour of the complex compound mixture within the extract, including the minor metabolites that we were not able to trace in the HPLC isolation process.

The antibacterial screening against strain Pa indicated that the total co-culture extract showed weak antibacterial effects, and the other pure compounds, except **5**, did not exhibit any activity within the test concentration series (Table 3), (Figure 5C). Compound **5** possessed a moderate antibacterial activity against strain Pa with MIC of 93 μM, which is three-fold lower than the positive control amoxicillin with MIC at 31 μM.

## 4. Discussion

Natural selection and environmental adaptation lead to successive generations of bacteria and fungi that acquire diverse defensive metabolites, ecologically and biologically potent natural products, encoded within a myriad of biosynthetic gene clusters (BGCs), to enable their survival in such competitive ecosystems [25]. For decades, microbial natural products have inspired new pharmaceuticals and agrochemicals, which are clearly exemplified in modern antibiotics derived from microbial natural products. Nevertheless, such success is challenged by exhausted microbial resources. In the last two decades, the challenge of drug resistance has emerged, leading to a search for new drug candidates. Recent advances in genetic engineering have indicated that there is a strong case that microbial biodiscovery could have a prominent role, due to their limitless reservoir of transcriptionally inactive (i.e., cryptic or silent) BGCs, dominant in all microbial genomes [26]. Recently, a range of approaches have proved promising for the activation of otherwise silent BGCs. One of the vigorous and cost-effective methods for the activation of silent BGCs in the producing strain is microbial co-cultivation. Microbial co-culture, or so-called mixed fermentation, has demonstrated a powerful strategy for mimicking the natural microbial communities to elicit the production of hitherto unexpressed chemical diversity through the induction of cryptic biosynthetic pathways to produce new SMs that were not traced in axenic cultures [4,5]. Competition for limiting natural resources is believed to be one of the major forces that promotes the activation of otherwise silent BGCs’ encoding for defensive secondary metabolites [27]. Another potential hypothesis for the stimulation of such silent SMs is microbial crosstalk via the production of signalling molecules, such as quorum sensing molecules and siderophores, in their environment [28,29].

In our initial confrontation screening, using a selection of diverse bacterial and fungal endophytes recovered from Algerian plants, co-culturing of the endophytic bacterial Pa and fungal Pc has shown a challenging interaction between the bacterial species and the fungal species by suppressing its propagation. This has triggered us to investigate the chemical profile differences between axenic and co-cultures. Initially, small-scale fermentation of bacterial and fungal axenic cultures and their co-culture was performed, and their chemical profiles were analysed by LC-HRMS. Interestingly, distinct chemical profiles were further elaborated by performing an extensive dereplication process using MZmine and different natural product databases. Dereplication of the co-culture extract showed a series of sulfur-containing metabolites, including few new hits. This inspired us to perform large-scale co-cultivation followed by chromatographic purification and spectroscopic characterisation of the isolated compounds. This process resulted in the identification of two new sulfur-containing metabolites (2 and 3), which were assigned as the pulicatin analogues pulicatin H and I, respectively, together with known compound **1**, aeruginaldehyde, which was previously isolated from *Pseudomonas fluorescens*; compound **4**, pulicatin F which was previously synthesised although this is its first isolation from a natural source; compound **5**, the fungal hexapeptide siderophore desferrichrome which was previously reported from the rust fungus *Ustilago sphaerogena*; two known cyclic dipeptides, 6 and 7, of bacterial and fungal origin, respectively; and, finally, compound **8**, 2-heptyl-4-hydroxy-quinolone (HHQ), which was reported as a bacterial metabolite from a marine sponge-derived *Pseudomonas* sp. and its molecular formula was confirmed in *Pantoea agglomerans* strain Pa during our metabolomics studies under OSMAC conditions [10].

Previous researches clearly indicate that the fungal metabolite pattern is usually affected in fungal–bacterial mixed fermentations, and no clear induction of bacterial cryptic metabolites is observed [6]. Recently, our group demonstrated the very first case reporting the dual induction of both silent bacterial and fungal metabolites through the co-cultivation of the marine-derived fungal isolate *Aspergillus fumigatus* MR2012 with the hyper-arid desert bacterial isolate *Streptomyces leeuwenhoekii* strain C34 [5]. The current study represents the second example of such a rare case of the expression of otherwise cryptic bacterial biosynthetic pathways to induce a lot of metabolites of different chemical classes, some proved through LC-HRMS analysis and some through large-scale co-cultivation and isolation of these major metabolites to assess their bioactivity and trying to find a clue regarding their expression.

Studying the literature of the readily known metabolites provided a cue for the initial fungal suppression in the confrontation screening, through either the induction of potentially antifungal metabolites or the production of siderophores or quorum-sensing molecules. Aeruginaldehyde 1 was reported to exhibit a potent biocontrol agent against various phytopathogenic fungi, such as *Cladosporium* spp. and *Fusarium* spp. [17,18]. Sulfur-containing bio-chemicals are available in all organisms in forms of primary as well as in SMs. In the cell, the main source of the sulfur atoms participating in the biological processes is the amino acid cysteine. Cephalosporins, a class of β-lactam antibiotics first reported from the fungus *Acremonium chrysogenum* [30], and the antitumor agent sparsomycin first isolated from *Streptomyces spursogene* [31], are two of numerous examples of naturally occurring and pharmaceutically important sulfur-containing metabolites. A series of neuroactive pulicatins A-E derivatives in addition to other structurally related analogues aerugine and watasemycins A and B were isolated from cone snail-associated *Streptomyces* sp. [19], which are recently considered to be siderophores [32]. Siderophores are identified as chemicals produced by the microorganisms to import iron for interspecies competition and play a crucial role in bacterial iron homeostasis [33]. Iron-containing enzymes play vital roles in living organisms. However, under natural environmental conditions, iron is present as an insoluble ferric hydroxide complexes, which limits its bioavailability. To overcome iron inaccessibility, microorganisms tend to produce high-affinity iron chelators, termed siderophores, that can solubilize iron and deliver it into the cells [34]. The literature reported the main function of the fungal metabolite desferrichrome 5 as the absorption of the inaccessible Fe(III) and the delivery of it into the microbial cells during competition with other species [34,35]. Therefore, it could be postulated that the production of 5 was triggered by the inter-species competition for food in the mixed fermentation environment. The LCMS profiles indicated that desferrichrome 5 was produced in trace amounts in the axenic fungal Pc culture and its production titre was increased in the co-cultivation experiment. Hence, we could postulate that compounds **1**–**5** act as siderophores which play an important role in bacterial iron homeostasis by acquiring iron from the surrounding environment, act as signalling molecules, regulate oxidative stress, and exhibit antimicrobial activity. These properties would rationalise their induction in the co-culture by creating a challenging environment for the other species.

Many Gram-negative bacteria proved to communicate via the production of small, diffusible signal molecules that organise virulence production and control the expression of genes responsible for communal behaviour and inter-species crosstalk, which is known as quorum sensing [36]. It is proved that 2,5-diketopiperazines or cyclic dipeptides are able to activate or inhibit quorum sensing in certain Gram-negative bacteria [36,37]. Therefore, it could be hypothesised that the isolated cyclic dipeptides 6 and 7 could have a quorum-sensing role in the crosstalk between the two microbial species. Additionally, compound **8**, HHQ is a hydroxyl quinoline analogue that has been previously identified as a product of Pa [10]. HHQ was identified as the direct precursor of the quorum-sensing molecule 2-heptyl-3-hydroxy-4(1H)-quinolone, also known as the pseudomonal quinolone signal (PQS) [38]. It was found that HHQ acts as an autoinducer molecule that is produced by *P. aeruginosa* cells and then taken up by neighbouring cells, which convert it into PQS [38]. In addition to the role of quorum-sensing molecules discovered in this study, antimicrobial screening of the expressed bacterial siderophore compounds **1**–**4** demonstrated their potential antifungal activities against Pc and *A. niger*. In the meantime, moderate antibacterial activity of the upregulated fungal siderophore compound **5** explained the attempt of Pc to withstand the challenging environment created by Pa in the mixed fermentation flask.

The fungal endophytes *Penicillium citrinum* isolate TDPEF34 used in this study was originally isolated from healthy date palm tree leaves and proved effective in confrontation assays against three pathogenic bacteria—*Bacillus* sp., *Enterococcus* sp., and *Salmonella* sp.—and phytopathogenic fungi—*Fusarium* sp. and *Trichoderma* sp.—due to its active secondary metabolites and volatile oil content [11]. On other occasions, *Penicillium citrinum* isolate IR-3–3 proved to possess a plant growth promoting ability, resulting in the maximum plant growth when applied to waito-c rice and *Atriplex gemelinii* seedling,s due to its gibberellins-producing ability [39]. The endophytic fungus *Penicillium citrinum* DBR-9 isolated from the root tubers of the Chinese plant *Stephania kwangsiensis* exhibited an in vitro antifungal effect on the hypha growth of tested plant pathogenic fungi [40]. In a study to investigate the plant growth promotion and stress mitigation effects of different *Penicillium* sp. on *Sesamum indicum* L., increased chlorophylls, proteins, amino acids, and lignans content were reported in addition to a significant increase in shoot length and fresh and dry seedling weights under salt stress conditions, and antagonistic activity toward the pathogenic fungi *Fusarium* spp. This study suggested that *Penicillium* sp. NICS01 can act as a biofertilizer and a biocontrol agent [41]. Moreover, *Penicillium citrinum* BTF08 was shown to have biocontrol potential against the phytopathogen *Fusarium oxysporum* f. sp. *cubense* in banana plantlets via induction of host resistance [42]. However, the only contradiction to these mentioned benefits for *Penicillium* was recently reported when *Penicillium citrinum* was identified as a new phytopathogen of orange fruit, which was inhibited using chitosan as an antifungal agent [43]. Although *Pantoea agglomerans* is an environmental Gram-negative bacterium associated with plants as an epi- or endophytic symbiont and exhibited promising biocontrol agents for a variety of bacterial and fungal plant diseases, particularly fire blight of apple and pear; it was recently reported with plant-pathogenic manifestations [44]. The bacterial endophyte *Pantoea agglomerans* strain Pa used in this study was originally isolated from durum wheat *Triticum durum* L., from the arid region Bou-Saâda in the South of Algeria. The study confirmed that *P. agglomerans* strain Pa possesses Plant Growth-Promoting Rhizobacterial characteristics, endorsing its implementation as an efficient biofertilizer in arid and salinity-impacted regions [10]. 

## 5. Conclusions

In conclusion, microbial co-culture (also referred to as mixed fermentation) proved to be a cheap and efficient approach to induce new microbial secondary metabolites. Although, in recent years, various interactions between bacteria and fungi have been investigated, the current study describes a rare example of the induction of otherwise cryptic bacterial biogenetic pathways. The newly induced bacterial siderophores proved to exhibit potential antifungal effects against both *Penicillium citrinum* isolate TDPEF34 (Pc) and *Aspergillus niger* IMI51433. This is the first report of pulicatin derivatives as antifungal agents, which opens the door for future in-depth research of this structural motif for the discovery of new antifungal agents. 

## Figures and Tables

**Table 1:** NMR spectroscopic data for compounds **1**–**4** (DMSO-*d*_6_, 600 MHz at 298 K).

Atom	1		2		3		4	
	*δ*_C_, mult.	*δ*_H,_ mult. (*J* in Hz)	*δ*_C_, mult.	*δ*_H,_ mult. (*J* in Hz)	*δ*_C_, mult.	*δ*_H,_ mult. (*J* in Hz)	*δ*_C_, mult.	*δ*_H,_ mult. (*J* in Hz)
1	185.1 (CH)	10.02, s	30.5 (CH_3_)	2.16. s	26.7 (C)	2.37, s	163.3 (C)	
2	153.5 (C)	-	206.5 (C)		198.7 (C)		149.4 (C)	
3	131.9 (CH)	8.72, s	51.2 (CH_2_)	a. 2.84, dd (15.3, 8.2) b.2.91, dd (15.7, 4.6)	135.3 (CH)	7.64, d (16.9)	124.4 (CH)	8.19, s
4			66.3 (CH)	5.17, q	127.1 (CH)	6.93, d (17.1)		
5	164.3 (C)	-	158.6 (C)		149.6 (C)		163.0 (C)	
6			114.9 (CH)	7.48, s	124.2 (C)	8.03, s		
8			164.8 (C)		164.9 (C)			
1′	118.6 (C)	-	119.1 (C))	-	119.5 (C)		116.3 (C)	-
2′	127.9 (CH)	8.19, dd (6.1, 1.8)	127.1 (CH)	8.00, dd (7.8, 1.7)	126.7 (CH)	8.17, dd (8.1, 2.8)	128.4 (CH)	8.29, dd (8.1, 3.0)
3′	116.9 (CH)	7.07, dd (7.5, 1.4)	119.8 (CH)	6.91, t (6.9)	118.4 (CH)	6.80, t (7.1)	118.5 (CH)	6.84, t (7.9)
4′	132.1 (CH)	7.37, dd (7.4,1.3)	131.2 (CH)	7.29, t (7.6)	131.9 (CH)	7.28, t (7.5)	131.8 (CH)	7.26, t (7.1)
5′	120.0 (CH)	6.99, t (7.5)	117.3 (CH)	7.02, d (8.7)	117.1 (CH)	6.98, d (9.1)	118.7 (C)	6.89, d (8.7)
6′	155.7 (C)	-	155.4 (C)		152.0 (C)		155.1 (C)	

**Table 2:** Chemical shift difference of (*S*)–MTPA and (*R*)–MTPA ester of compound **2**.

C/H No.	Chemical Shift (^1^_H_ in pyridine–*d*_5_ at 500 MHz)			
	5.17	(*S*)–MTPA Ester	(*R*)–MTPA Ester	∆*S*–*R*
4	5.453	5.455	5.815	−0.36

**Table 3:** In vitro Antimicrobial results of compounds **1**–**8** against Pc and Pa.

Compound	MIC (μM)			
	Pc	Pa	*A. niger*	*C. albicans*
**1**	43 (±1.6)	>200	40.2 (±1.2)	>50
**2**	25 (±1.1)	>200	8.4 (±0.7)	>50
**3**	127 (±1.3)	>200	12.8 (±0.8)	>50
**4**	53 (±1.5)	>200	22.6 (±0.3)	>50
**5**	>200	93 (±1.6)	>50	>50
**6**	>200	>200	>50	>50
**7**	>200	>200	>50	>50
**8**	>200	>200	>50	>50
Amphotericin B	16.7 (±1.2)		5.7 (±0.2)	2.4 (±0.1)
Amoxicillin		31 (±1.1)		

## Supplementary Materials

The following are available online at https://www.mdpi.com/2218-273X/10/2/268/s1. The 1D and 2D NMR data and HRMS analysis of the pulicatin derivatives are available at Figures S1–S21. Tables S1–S3 include the LC-HRMS dereplication analysis of Pa, Pc, and the co-culture extracts.
