# Supplementary material for: Induction of Cryptic Antifungal Pulicatin Derivatives from Pantoea Agglomerans by Microbial Co-Culture
Source: Biomolecules. 2020 Feb 10;10(2):268. doi: 10.3390/biom10020268 (PMC7072716; doi:10.3390/biom10020268)
Supplement: Supplementary file 1 [file biomolecules-10-00268-s001.pdf]

Supplementary Information

Induction of cryptic antifungal pulicatin derivatives from *Pantoea agglomerans* by microbial co-culture

Bathini Thissera,[1] Hani A. Alhadrami,[2,3,*] Marwa H.A. Hassan,[4] Hossam M. Hassan,[4] Fathy A. Behery,[5,6] Majed Bawzeer,[1] Mohammed Yaseen,[1] Lassaad Belbahri,[7] and Mostafa E. Rateb[1,4,*]

[1] School of Computing, Engineering & Physical Sciences, University of the West of Scotland, Paisley PA1 2BE, Scotland, UK,

[2] Department of Medical Laboratory Technology, Faculty of Applied Medical Sciences, King Abdulaziz University, Jeddah, 21589, Saudi Arabia,

[3] King Fahd Medical Research Centre, King Abdulaziz University, Jeddah, 21589, Saudi Arabia,

[4] Department of Pharmacognosy, Faculty of Pharmacy, Beni-Suef University, Beni-Suef, 62514, Egypt

[5] Department of Pharmacognosy, Faculty of Pharmacy, Mansoura University, Mansoura 35516, Egypt,

[6] Department of Pharmacy, College of Pharmacy, Riyadh Elm University, Riyadh 11681, Saudi Arabia,

[7] Laboratory of Soil Biology, University of Neuchatel, 2000 Neuchatel, Switzerland.

[*] **Correspondence: Mostafa E. Rateb: Tel +441418483072; email Mostafa.Rateb@uws.ac.uk; Hani A. Alhadrami: Tel +966505545275, email: hanialhadrami@kau.edu.sa**

Table S1. Pc LC-HRMS dereplication

Retention time (min)	m/z [M+H]+	Molecular formula	Tentative Identification	structure
3.46	197.1293	$C_{10}H_{16}N_2O_2$	cyclo(pro-val)	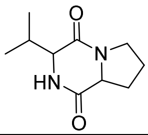
4.28	279.1159	$C_{17}H_{14}N_2O_2$	dehydrocyclopeptin	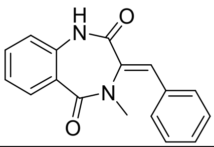
5.48	197.1285	$C_{10}H_{16}N_2O_2$	cyclo(Pro-Val)	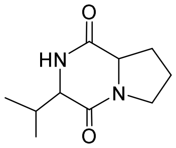
5.75	166.0865	$C_9H_{11}NO_2$	p-dimethylamino-benzoic acid	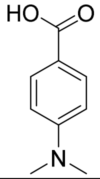
6.57	225.0880	$C_{10}H_{12}N_2O_4$	N-{3-Nitro-4-hydroxyphenylethyl} acetamide	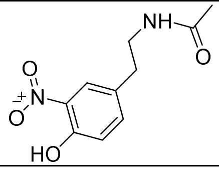

6.57	324.1566	$C_{17}H_{24}O_7$	AF toxin 11A	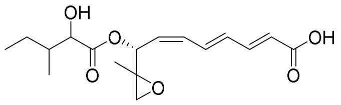
6.59	324.1566	C20H22NO	New hit	
8.17	254.0763	$C_{15}H_{11}NO_3$	phomopsin A	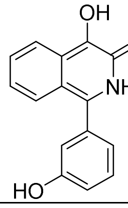
8.39	235.1446	$C_{13}H_{18}N_2O_2$	Fungerin	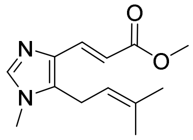
9.10	281.1289	$C_{17}H_{16}N_2O_2$	cyclopeptin	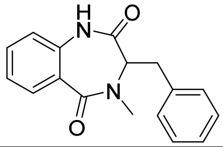
9.12	628.3254	$C_{22}H_{45}O_{12}N_9$	desferrichrome	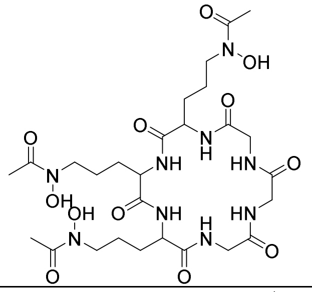
9.64	221.8219	$C_{12}H_{12}O_4$	Hispolon	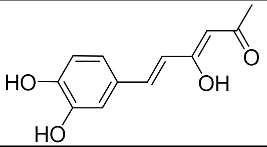
9.69	211.14454	$C_{11}H_{18}N_2O_2$	Cyclo(Pro-leu)	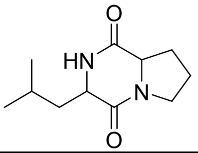
9.88	172.0427	$C_7H_9NO_2S$	4-methylbenzene sulfonamide	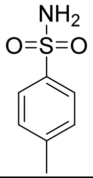
9.69	311.1394958	$C_{18}H_{18}N_2O_3$	4'-Methoxycyclopeptin	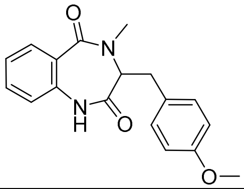
9.88	190.0323	$C_{10}H_7NOS$	New hit	

10.19	203.1433	$C_{14}H_{18}O$	Penidienone	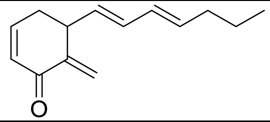
10.95	244.1701	$C_{16}H_{21}NO$	Isochalciporon	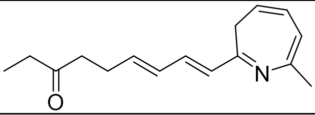
11.43	162.0549	$C_9H_7NO_2$	7-isocyano-1-oxaspiro[4.4]nona-3,6-dien-2-one	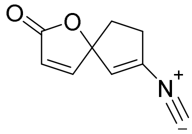
12.05	432.1598	$C_{22}H_{25}NO_8$	Pseurotin A	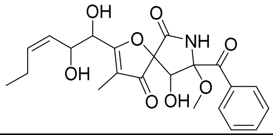
12.53	444.22818	$C_{27}H_{29}N_3O_3$	Fructigenine	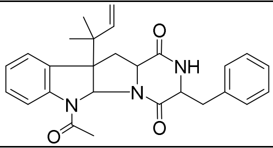
15.87	311.0954	$C_{17}H_{14}N_2O_4$	cyclopeniol	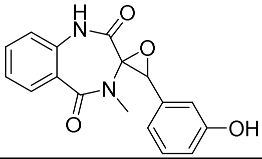
13.00	227.1018	$C_{11}H_{18}N_2O_3$	Cyclo(4-OH-pro-Leu)	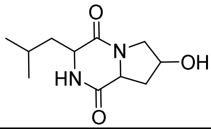
13.18	171.1380	$C_{10}H_{18}O_2$	5-Decanolide	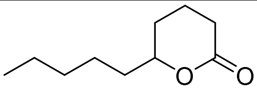
16.50	673.3775	$C_{30}H_{52}O_{11}N_6$	New hit	
17.81	1038.6356	$C_{68}H_{83}O_6N_3$	New hit	

Table S2. Pa LC-HRMS dereplication (Cherif-Silini *et al*, 2019)

Rt (min)	m/z $[M+H]^+$	Molecular formula	Tentative Identification	Structure
5.02	235.1082	$C_{12}H_{14}N_2O_3$	Cyclo(Phe-Ser)	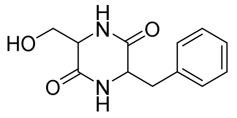
5.02	247.1083	$C_{13}H_{14}N_2O_3$	N-Acetyltryptophan	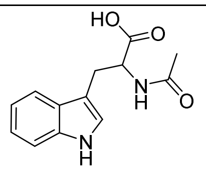
5.10	261.18533	$C_{14}H_{16}O_3N_2$	cyclo(Pro-Tyr)	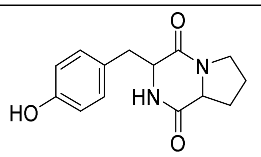
5.11	205.0976	$C_{11}H_{12}N_2O_2$	Cyclo(Gly-Phe)	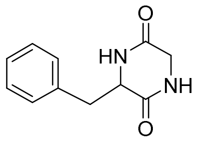
5.20	217.0977	$C_{12}H_{12}N_2O_2$	Sannanine	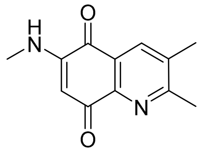
5.40	197.12887	$C_{10}H_{16}N_2O_2$	cyclo(Pro-Val)	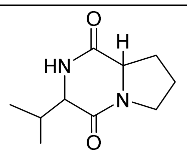
5.58	185.1287537	$C_9H_{16}N_2O_2$	cyclo(Ala-Leu)	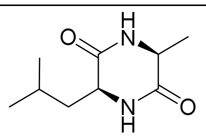
5.80	227.1395645	$C_{11}H_{18}N_2O_3$	cyclo-4-hydroxyPro-Leu	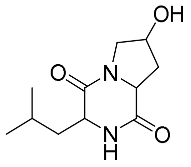

5.94	211.1446	$C_{11}H_{18}O_2N_2$	Cyclo(Ile-Pro)	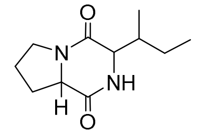
6.90	219.1133	$C_{12}H_{14}N_2O_2$	Cyclo(Ala-Phe)	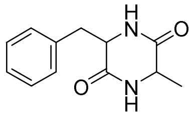
6.90	277.1551	$C_{15}H_{20}N_2O_3$	cyclo(Leu-Tyr)	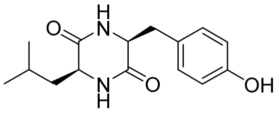
7.12	239.0820	$C_{14}H_{10}N_2O_2$	6-Carboxymethyl-phenazine	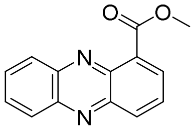
7.76	176.0708	$C_{10}H_9NO_2$	4-Hydroxy-N-methylcarbostyril	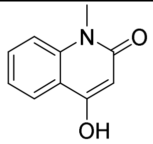
8.74	245.1289	$C_{14}H_{16}N_2O_2$	cyclo-(Pro-phe)	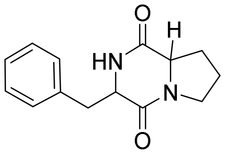
9.69	311.1394	$C_{18}H_{18}N_2O_3$	4'-Methoxycyclopeptin	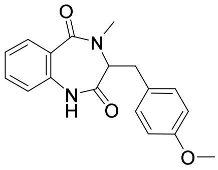
10.22	213.1602	$C_{11}H_{20}N_2O_2$	cyclo(L-Ile-L-Valine)	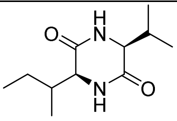
11.48	178.0324	C_9H_7NOS	2-(2-Hydroxyphenyl)thiazole	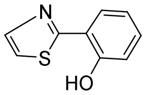
11.52	224.0381	$C_{10}H_9NO_3S$	(+)-(S)-dihydroaeruginoic acid	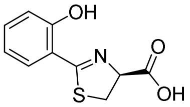
12.61	218.1182	$C_{13}H_{15}NO_2$	New quinoline derivative	
12.65	261.1603	$C_{15}H_{20}N_2O_2$	Cyclo(Leu-Phe)	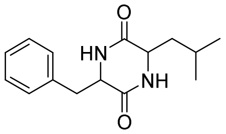

13.18	214.1232	$C_{14}H_{15}NO$	New quinoline derivative	
13.40	295.1448	$C_{18}H_{18}N_2O_2$	cyclo-(L-Phe-L-Phe)	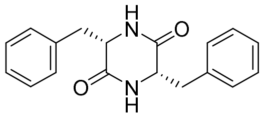
13.51	276.1602	$C_{16}H_{21}NO_3$	3-n-Heptyl-3-hydroxy-(1H,3H)-quinoline-2,4-dione	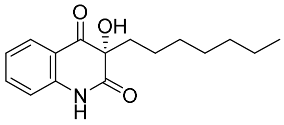
13.62	284.1651	$C_{18}H_{21}NO_2$	New quinoline derivative	
13.98	244.1713	$C_{16}H_{21}NO$	2-heptyl-4-hydroxy-quinolone	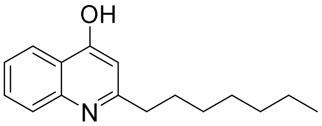
14.01	226.1231	$C_{15}H_{15}NO$	(E)-4-Phenyl-3-(pyridine-2-yl)but-2-en-1-ol	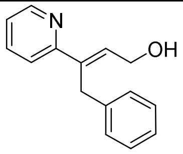
14.06	228.1389	$C_{15}H_{17}NO$	New quinoline derivative	
14.49	211.0872	$C_{13}H_{10}N_2O$	Cyanomycin	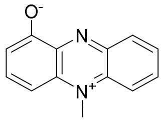
14.79	228.1389	$C_{15}H_{17}NO$	New quinoline derivative	
14.88	274.1444	$C_{16}H_{19}NO_3$	New quinoline derivative	
14.88	323.0530	$C_{14}H_{16}N_2O_3S_2$	Pyochelin	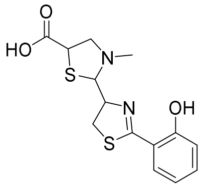
15.70	274.1809	$C_{17}H_{23}NO_2$	Lorneamide A	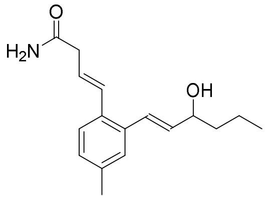
15.81	325.0679	$C_{14}H_{16}N_2O_3$	cyclo(4-OH-Pro-Phe)	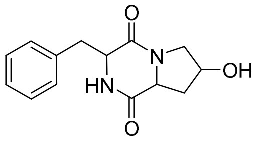
16.07	246.1494	$C_{15}H_{19}NO_2$	New quinoline derivative	

16.33	244.1338	$C_{15}H_{17}NO_2$	New quinoline derivative	
16.54	242.1548	$C_{16}H_{19}NO$	2-Heptenyl-4-quinolinone	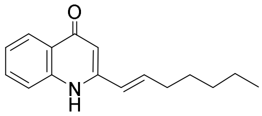
16.87	274.1444	$C_{16}H_{19}NO_3$	€-3-(hept-1-en-1-yl)-3-hydroxyquinoline-2,4(1H,3H)-dione	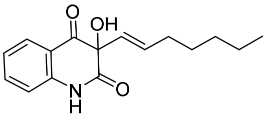
17.01	651.2722	$C_{30}H_{41}O_{13}N_3$	New hit	
18.32	260.1654	$C_{16}H_{21}NO_2$	2-Heptyl-3-hydroxy-4(3H)-quinolone	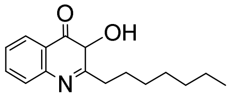
19.43	272.1645	$C_{17}H_{21}NO_2$	New quinoline derivative	
19.14	272.1647	$C_{17}H_{21}NO_2$	New quinoline derivative	
19.19	274.1805	$C_{17}H_{23}NO_2$	4-hydroxy-2-octylquinoline 1-oxide	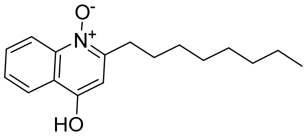
19.63	270.1861	$C_{18}H_{23}NO$	2-(n-delta(1)-Nonenyl)-4-oxyquinoline	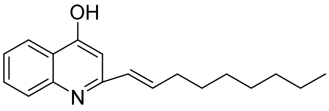
19.84	272.2017	$C_{18}H_{25}NO$	1. 2-Nonyl-quinonyl-4-ol	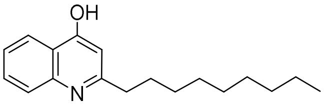
19.7	358.2380	$C_{22}H_{31}NO_3$	New quinoline derivative	
19.93	356.2224	$C_{22}H_{29}NO_3$	New quinoline derivative	
20.15	277.1172	$C_{16}H_{22}O_4$	Oncorhyncolide	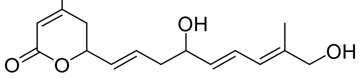
20.16	286.1809	$C_{18}H_{23}NO_2$	New quinoline derivative	
20.89	288.1965	$C_{18}H_{25}NO_2$	4-Hydroxy-2-n-nonanyl-quinoline N-oxide	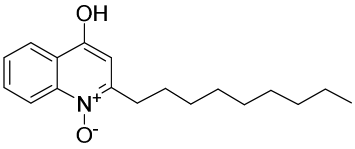

21.67	314.2118	$C_{20}H_{27}NO_2$	(Z)-4-hydroxy-2-(undec-4-en-1-yl)quinoline 1-oxide	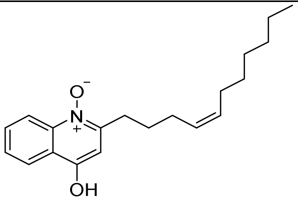
22.53	340.22730	$C_{22}H_{29}NO_2$	New quinoline derivative	

Table S3. Pa-Pc LC-HRMS dereplication

Retention time (min)	m/z [M+H]$^+$	Molecular formula	Tentative Identification	structure
5.41	197.1274	$C_{10}H_{16}O_2N_2$	cyclo(pro-Val)	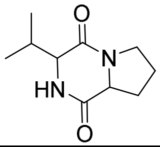
7.31	684.3877	$C_{30}H_{53}N_9O_7S$	New hit	
7.35	206.993	$C_{10}H_7NO_2S$	Aeruginaldehyde	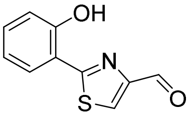
8.06	560.26715	$C_{22}H_{37}O_{10}N_7$	New hit	
9.15	628.3254	$C_{22}H_{45}O_{12}N_9$	Desferrichrome	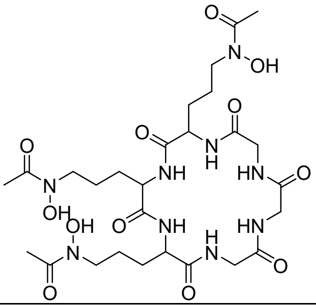
9.32	221.0385	$C_{10}H_8O_2N_2S$	Pulicatin F	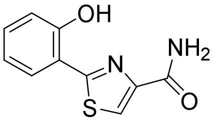
9.41	264.0695	$C_{13}H_{13}NO_3S$	New hit	
9.45	293.14984	$C_{15}H_{20}O_4N_2$	5Z-Bripiodionen	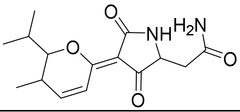

11.74	229.1552	$C_{11}H_{20}O_3N_2$	Libramycin A	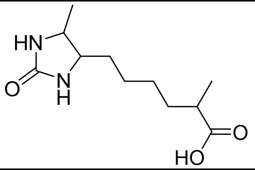
12.86	246.0519	$C_{13}H_{11}NO_2S$	New hit	
13.12	227.1018	$C_{11}H_{18}N_2O_3$	Cyclo(4-OH-pro-Leu)	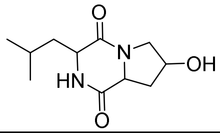
13.87	244.1713	$C_{16}H_{21}NO$	2-heptyl-4-hydroxy-quinolone	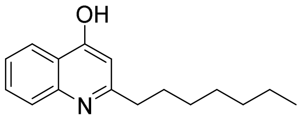

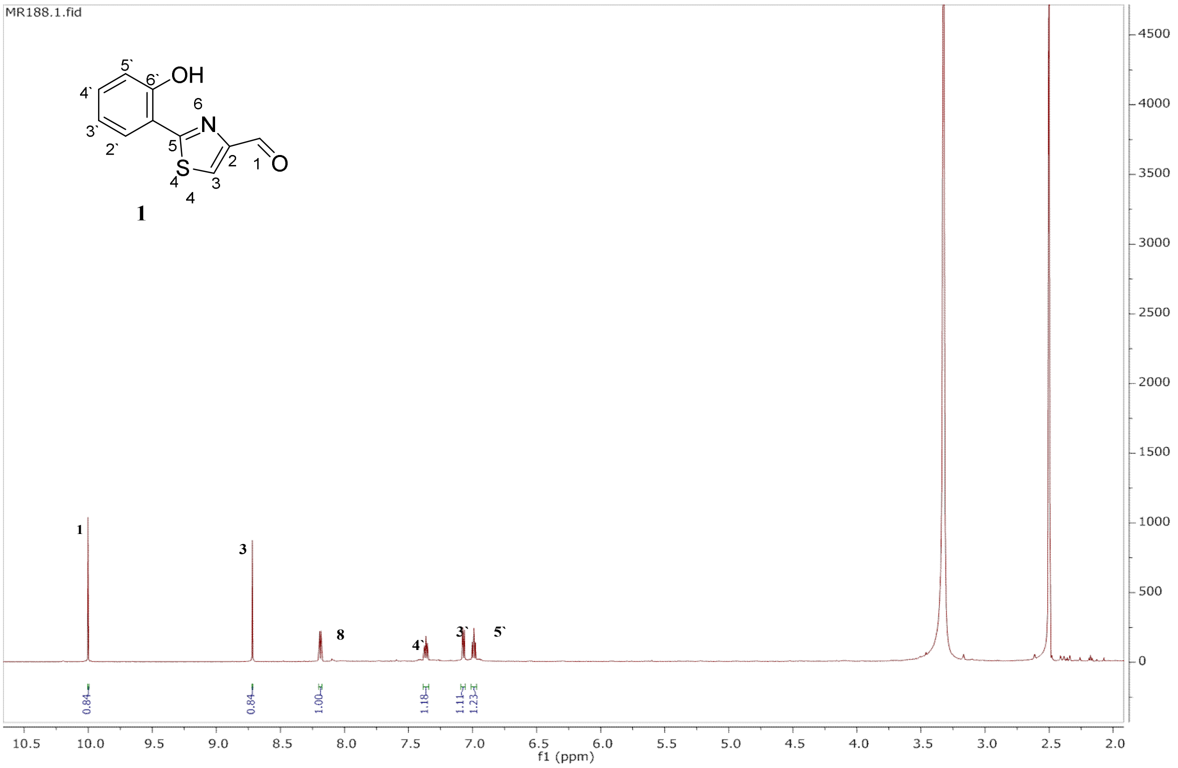

Figure S1. ^{1}H NMR spectrum of compound **1**.

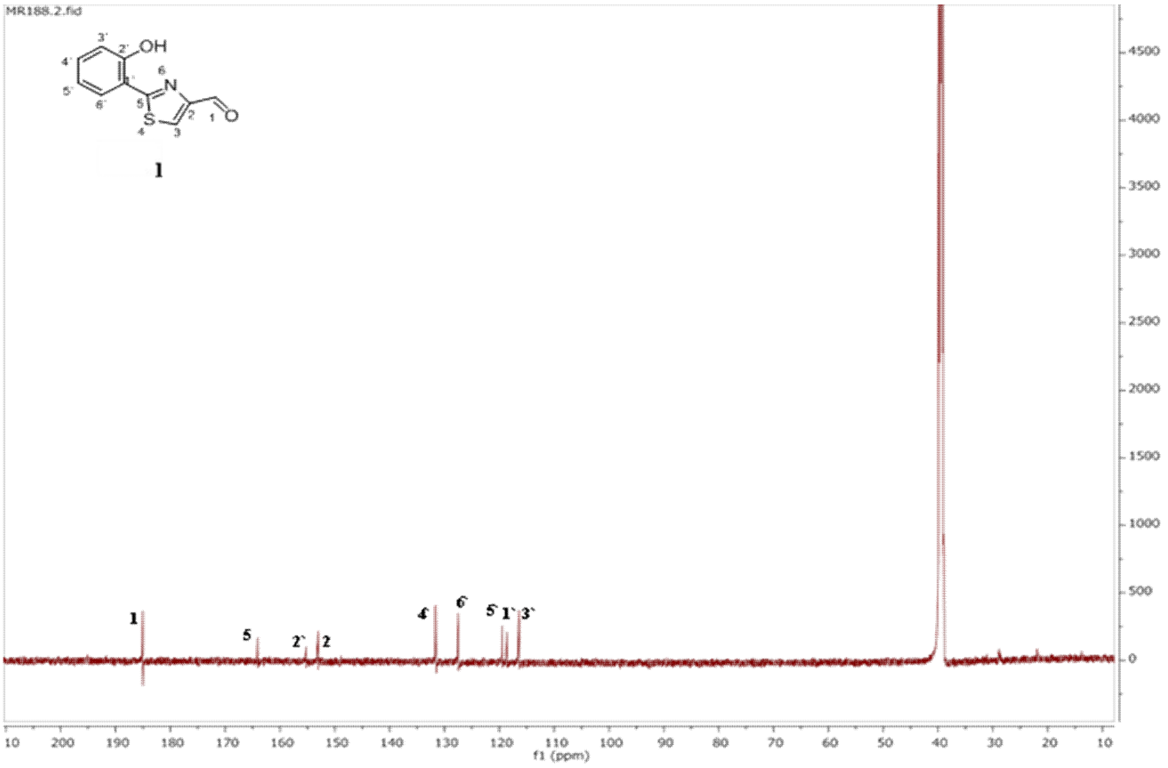

Figure S 2. ^{13}C NMR spectrum of Compound **1**.

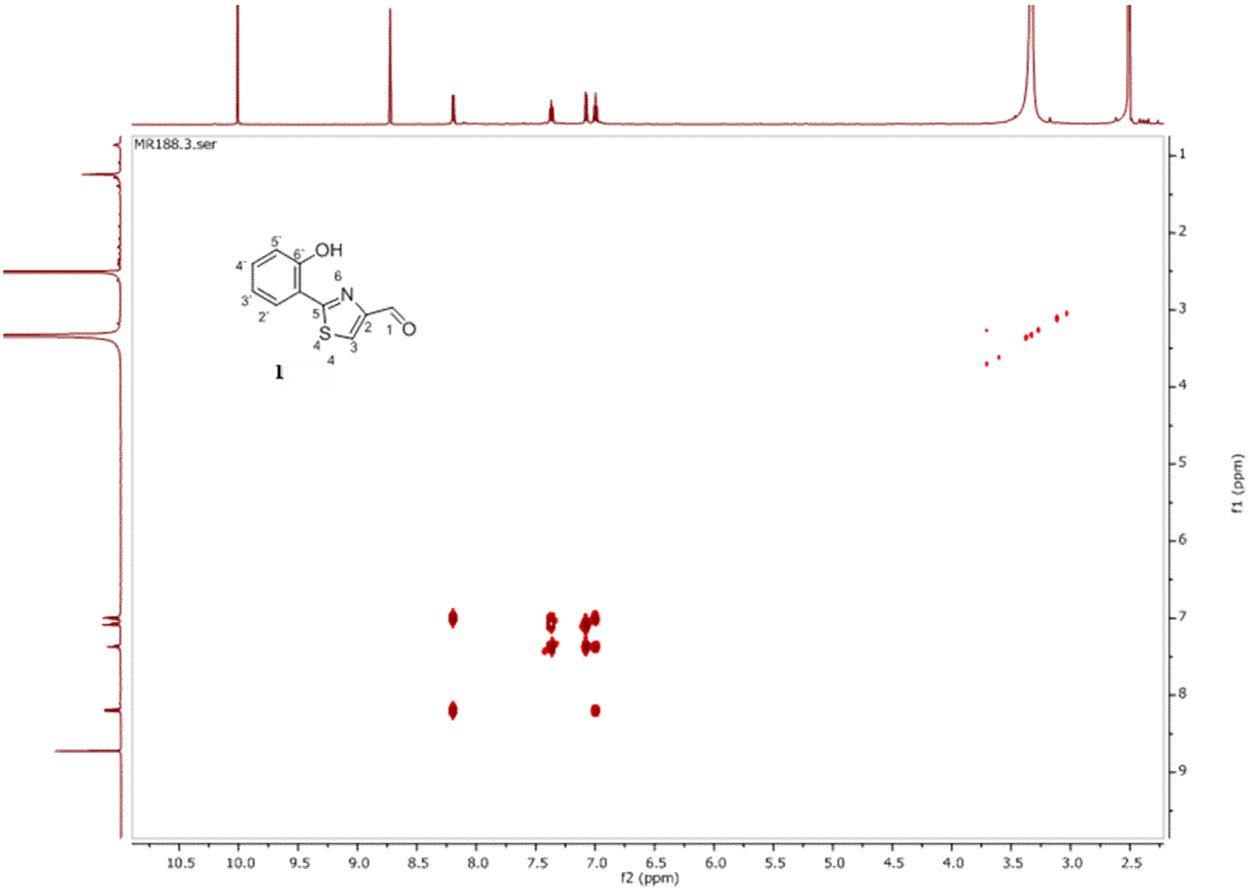

Figure S3. COSY spectrum of compound **1**

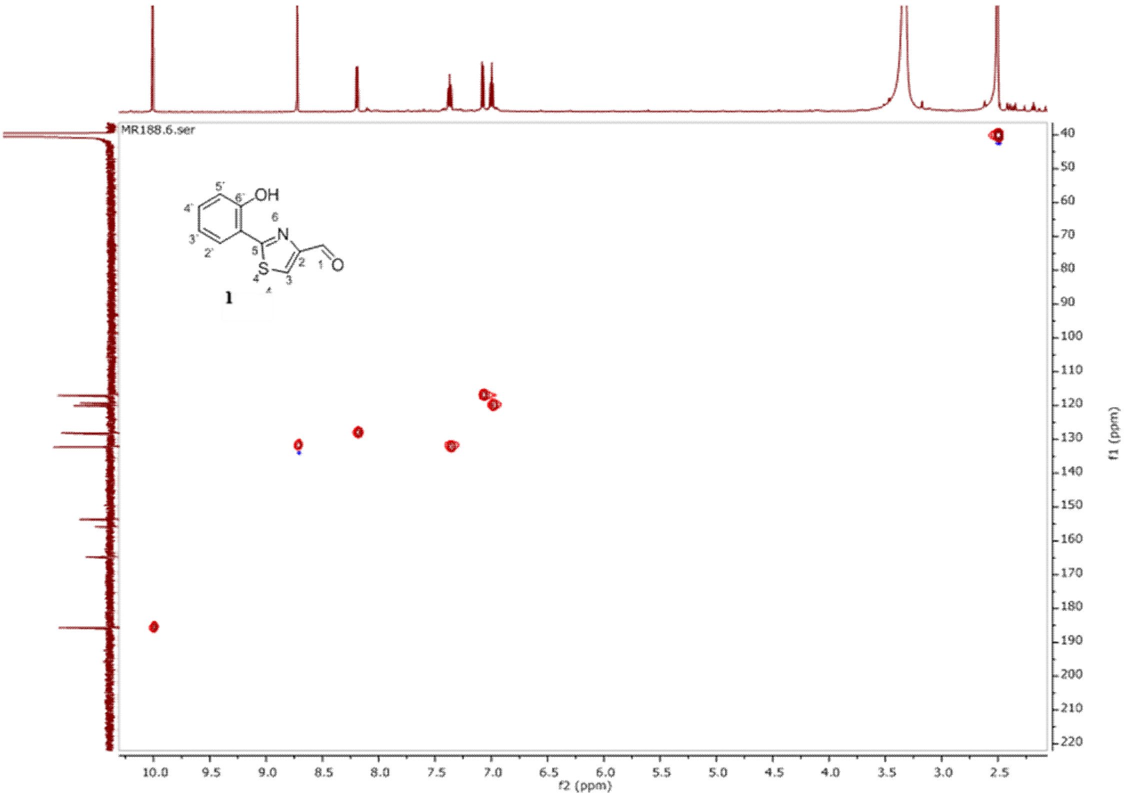

Figure S4. HSQC spectrum of compound **1**

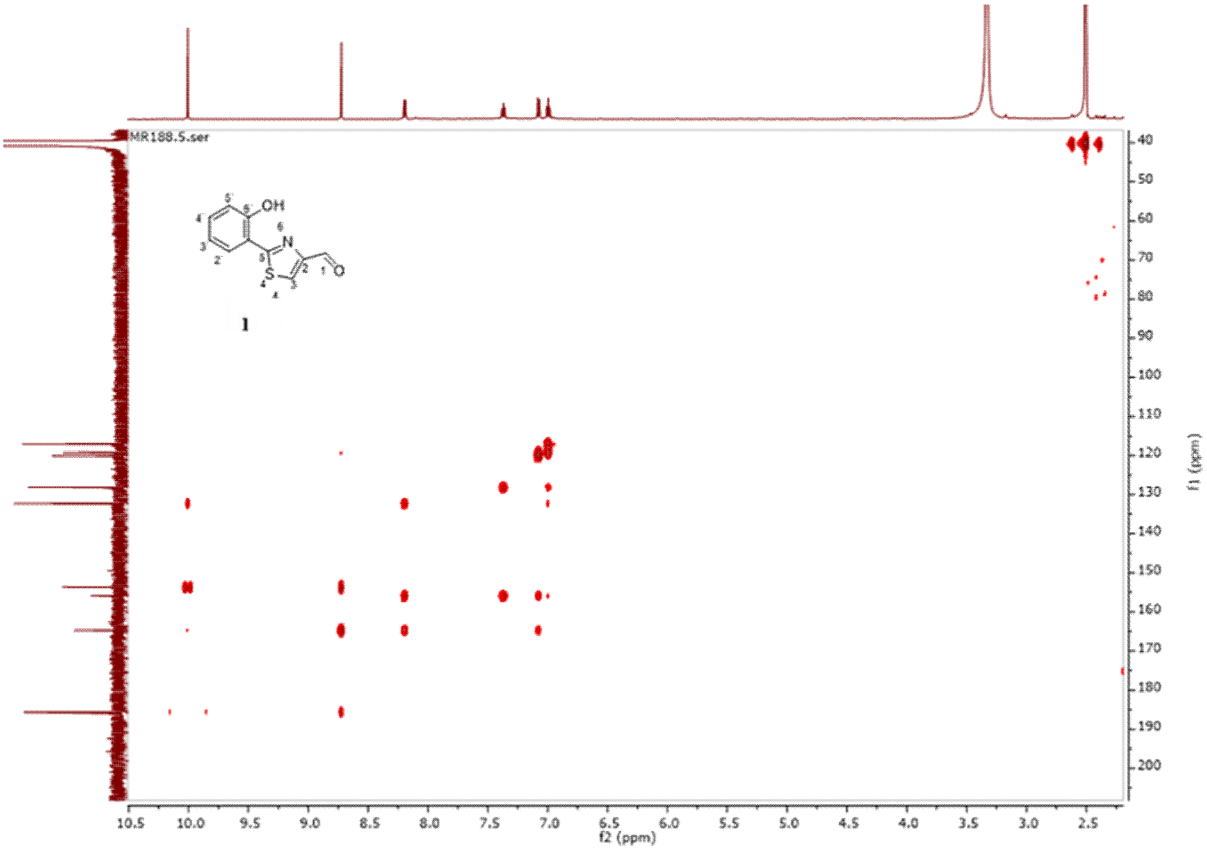

Figure S5. HMBC spectrum of compound **1**

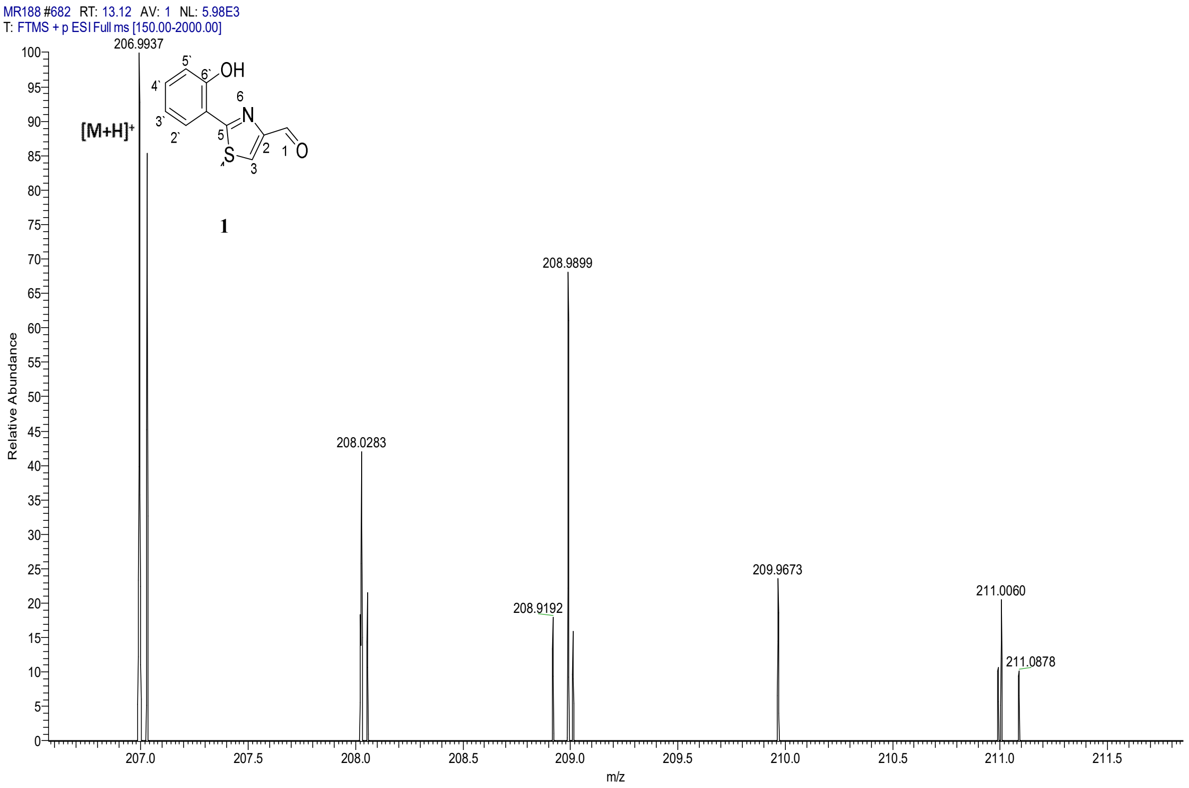

Figure S6. HRESIMS spectrum of compound **1**

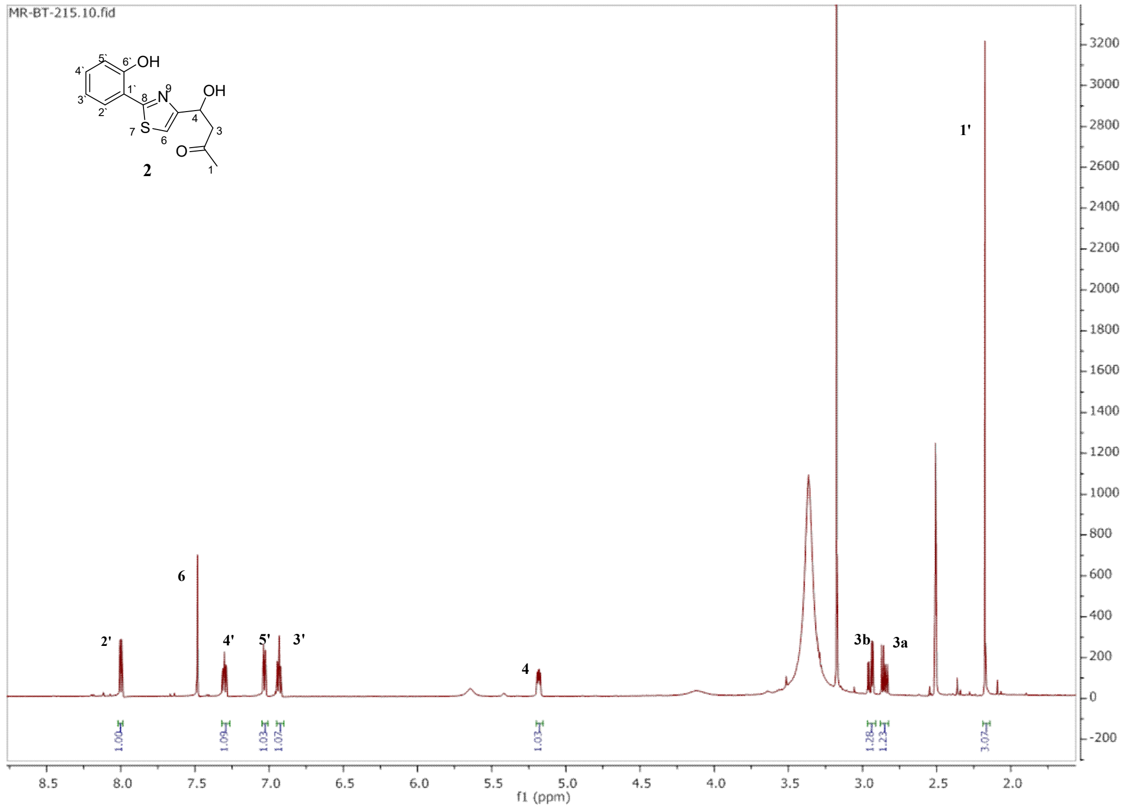

Figure S7. ^{1}H NMR spectrum of compound **2**.

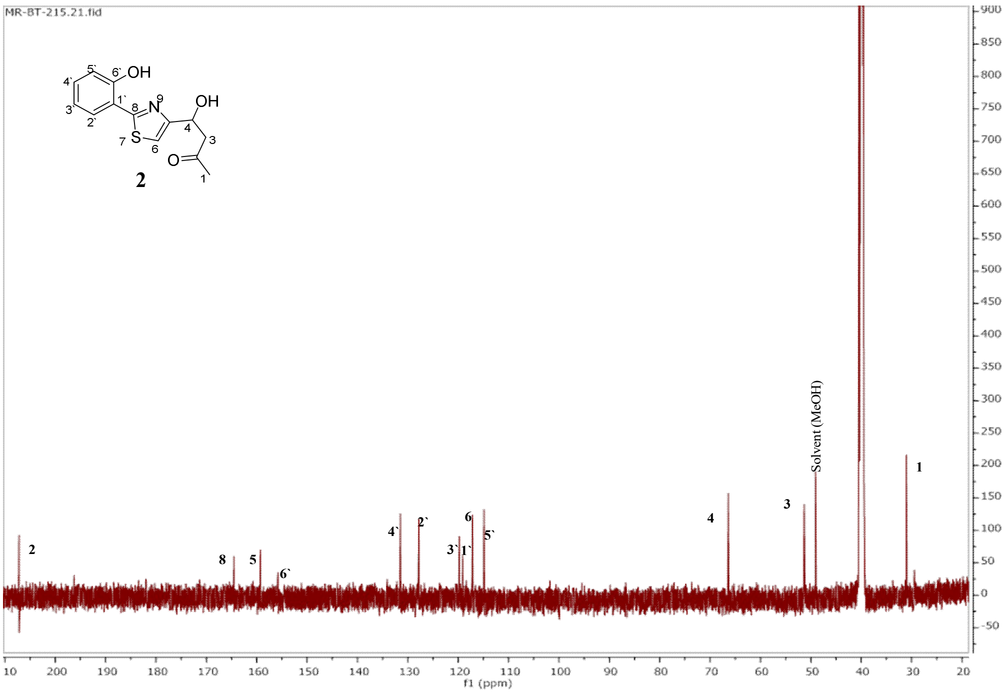

Figure S8. ^{13}C NMR spectrum of compound **2**.

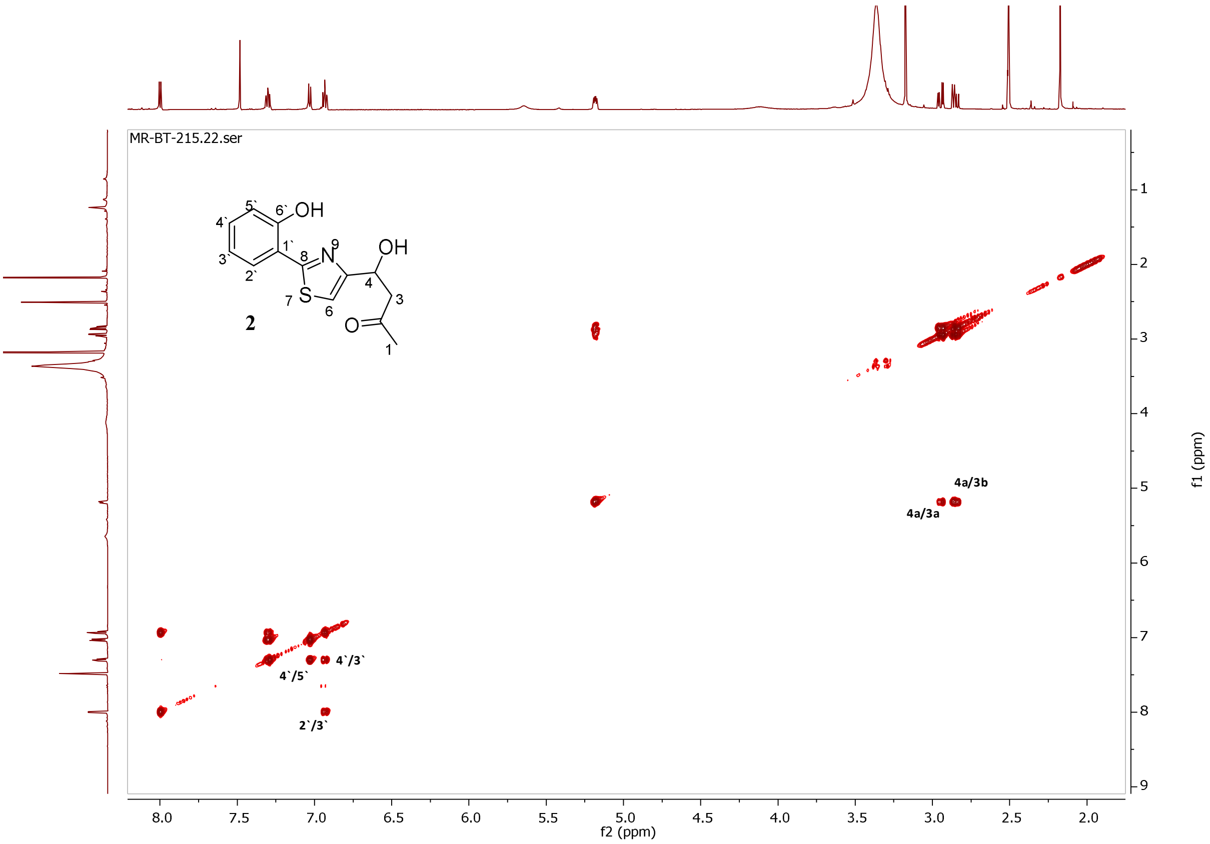

Figure S9. COSY spectrum of compound **2**

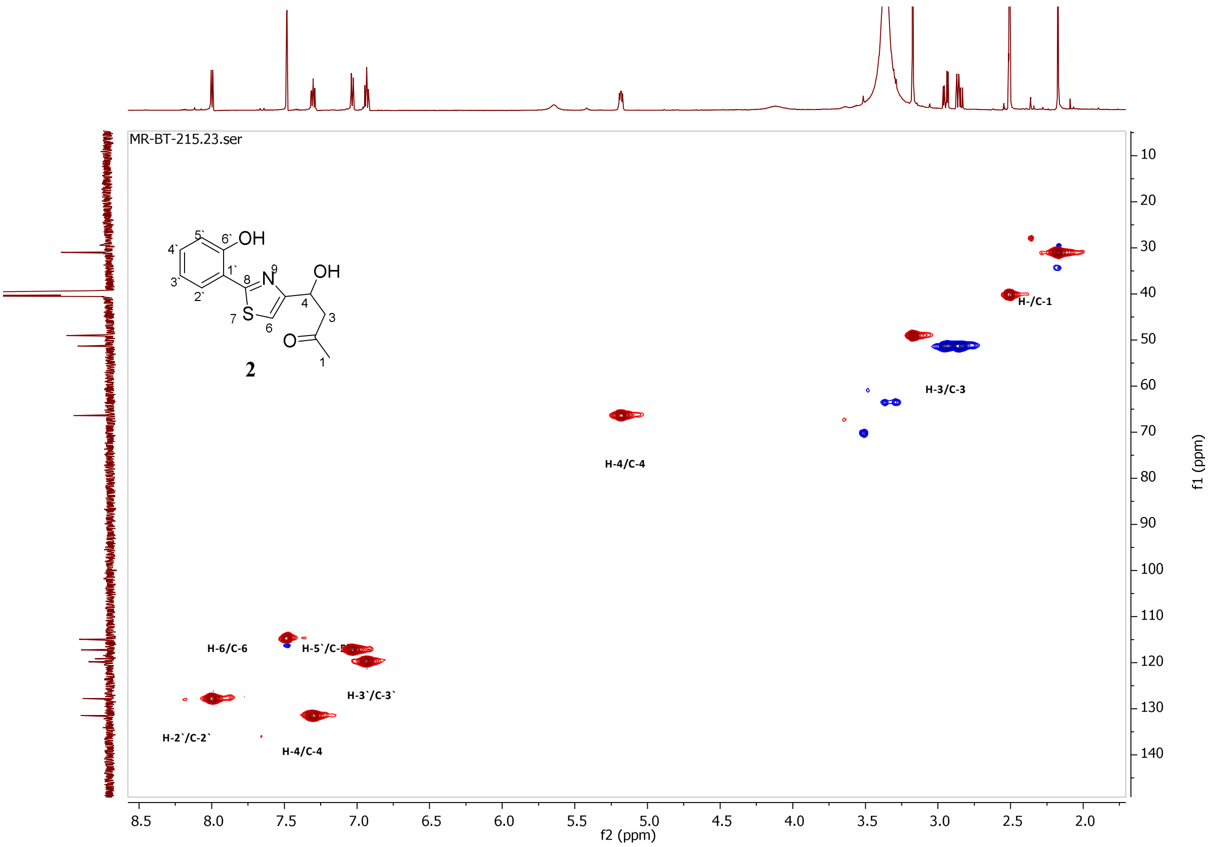

Figure S10. HSQC spectrum of compound **2**

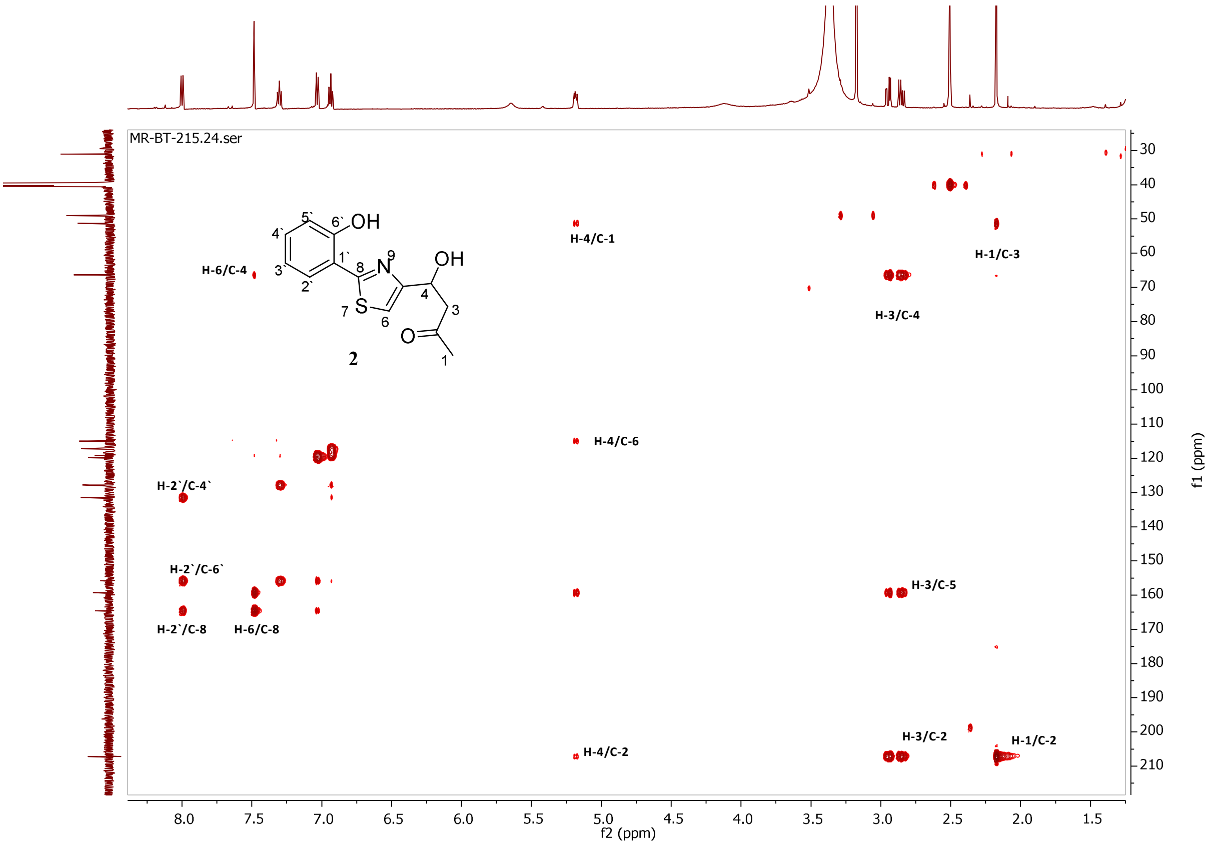

Figure S11. HMBC spectrum of compound **2.**

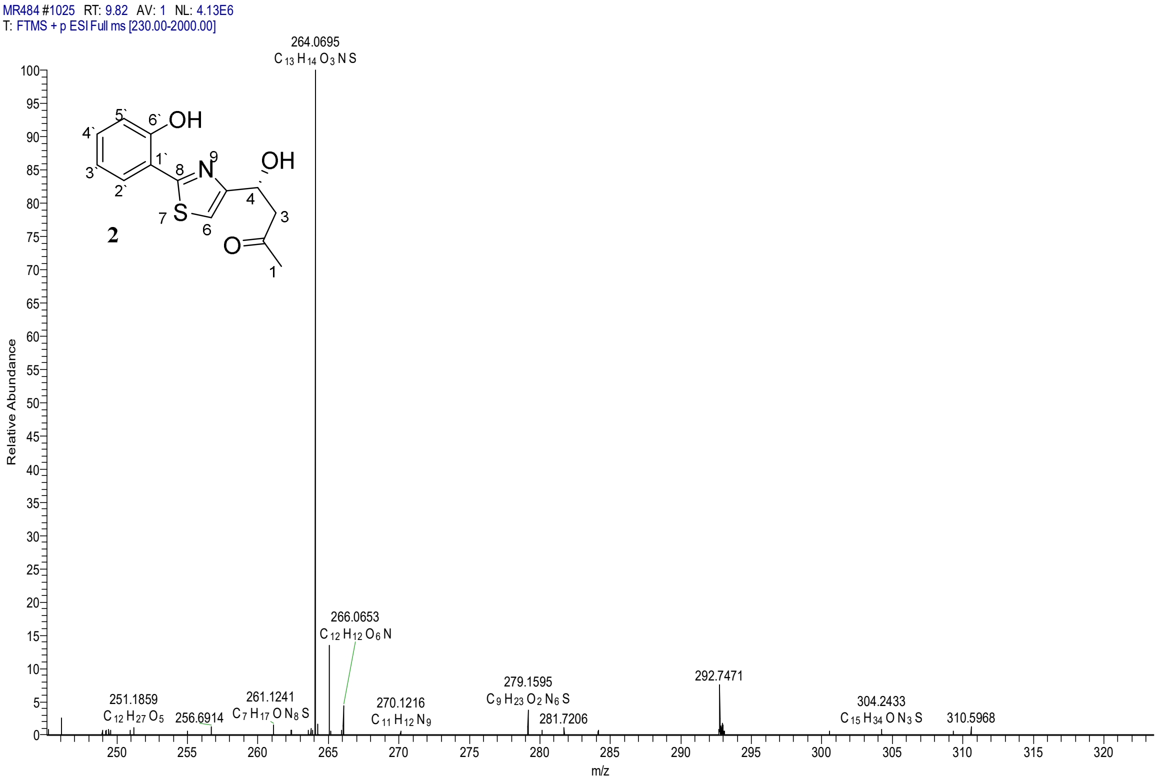

Figure S12. HRMS spectrum of compound **2.**

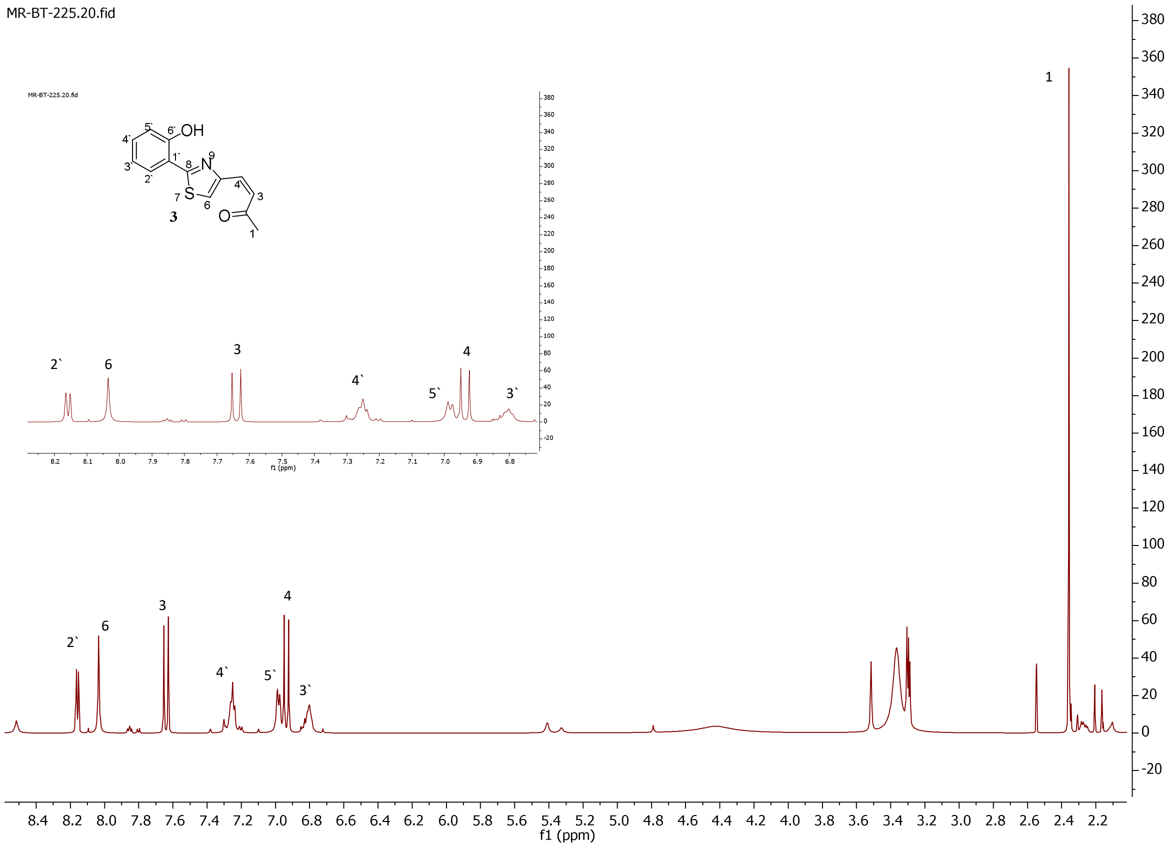

Figure S13. ^{1}H NMR spectrum of compound **3.**

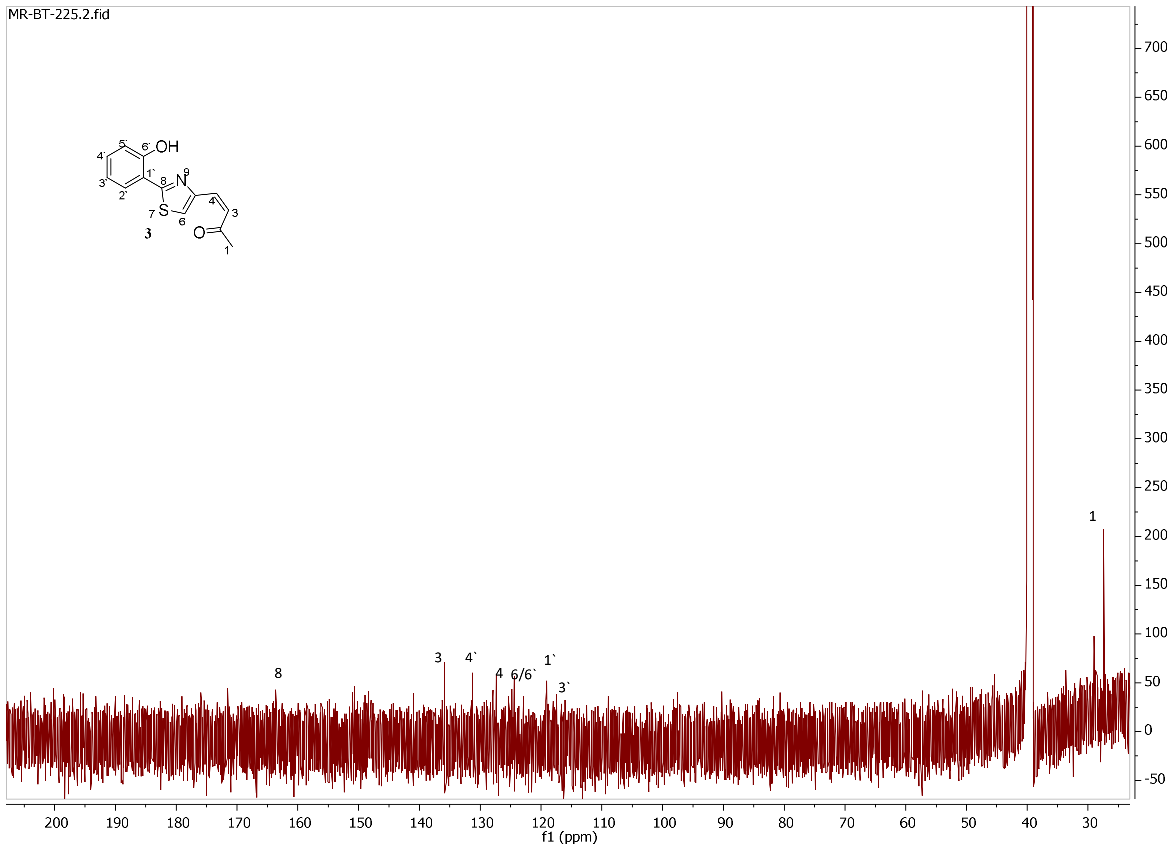

Figure S14. ^{13}C NMR spectrum of compound **3.**

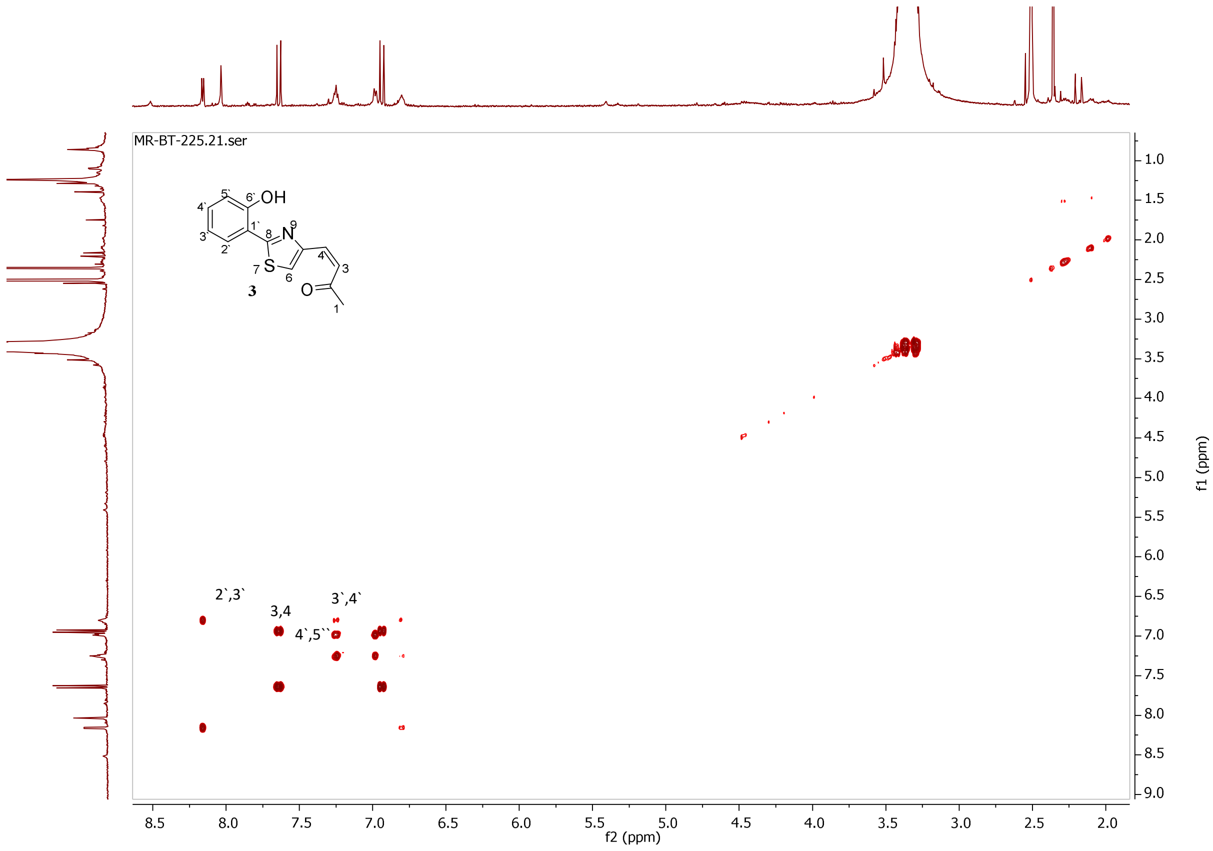

Figure S15. COSY spectrum of compound **3.**

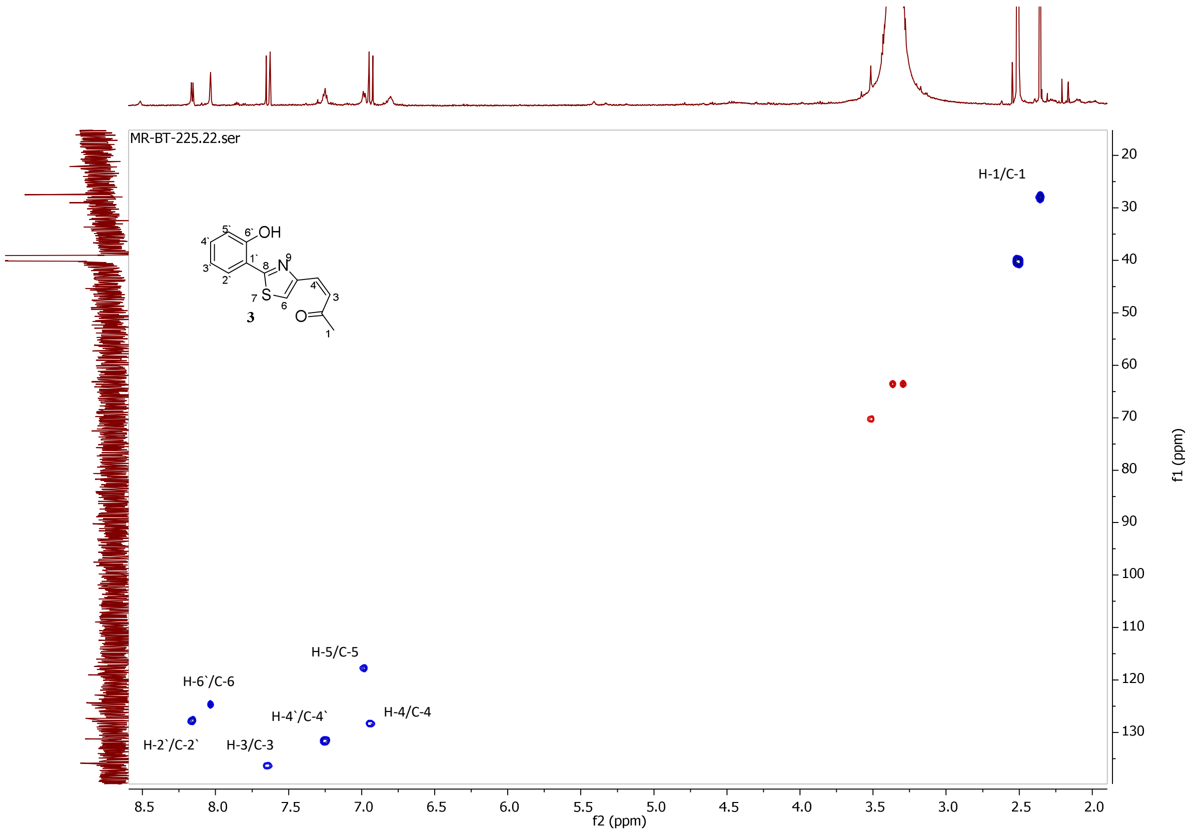

Figure S16. HSQC spectrum of compound **3.**

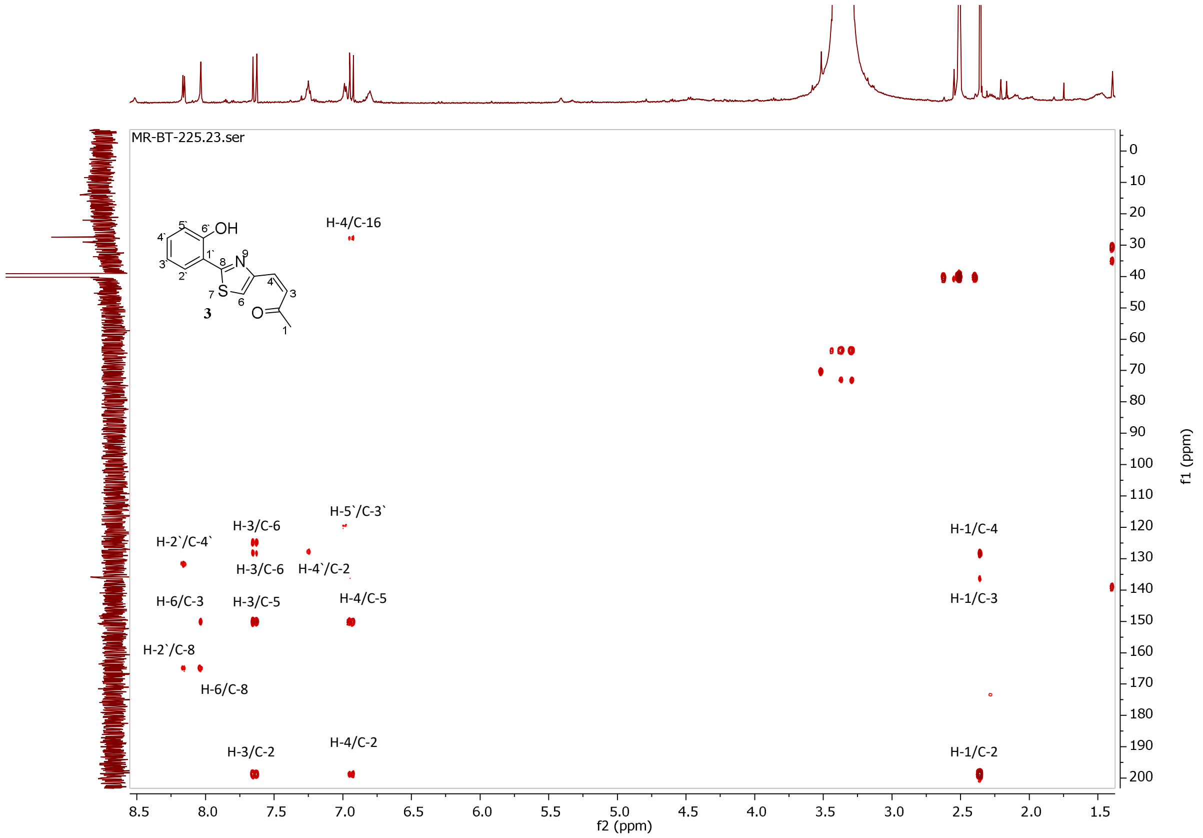

Figure S17. HMBC spectrum of compound **3.**

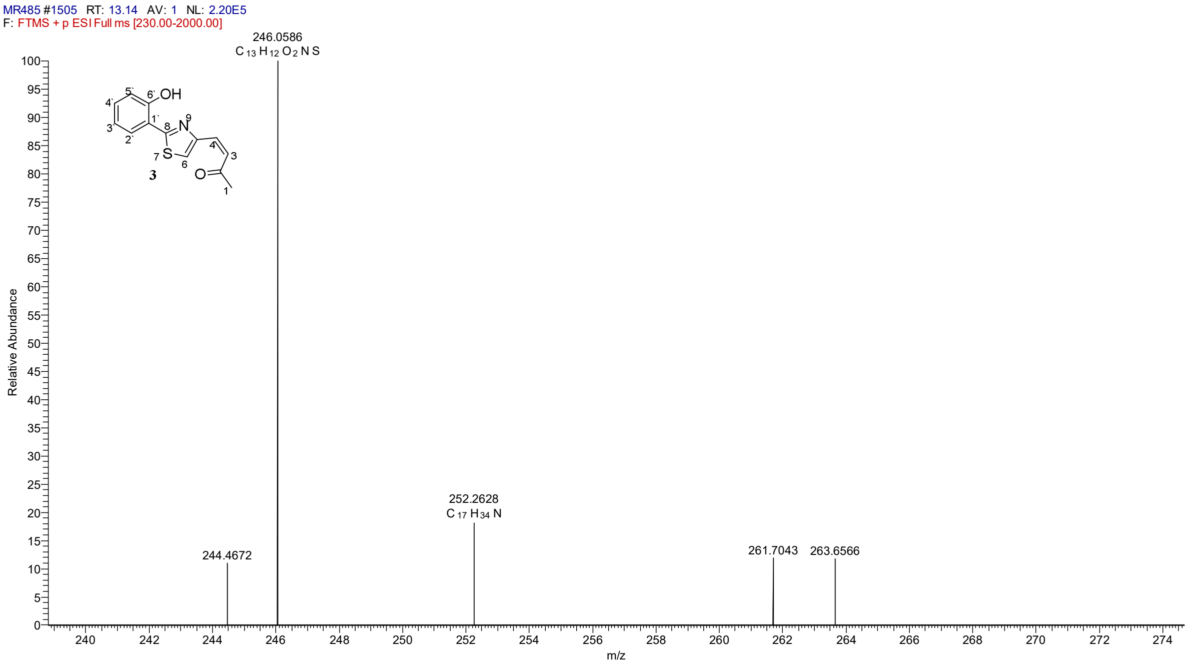

Figure S18. HRMS of compound **3.**

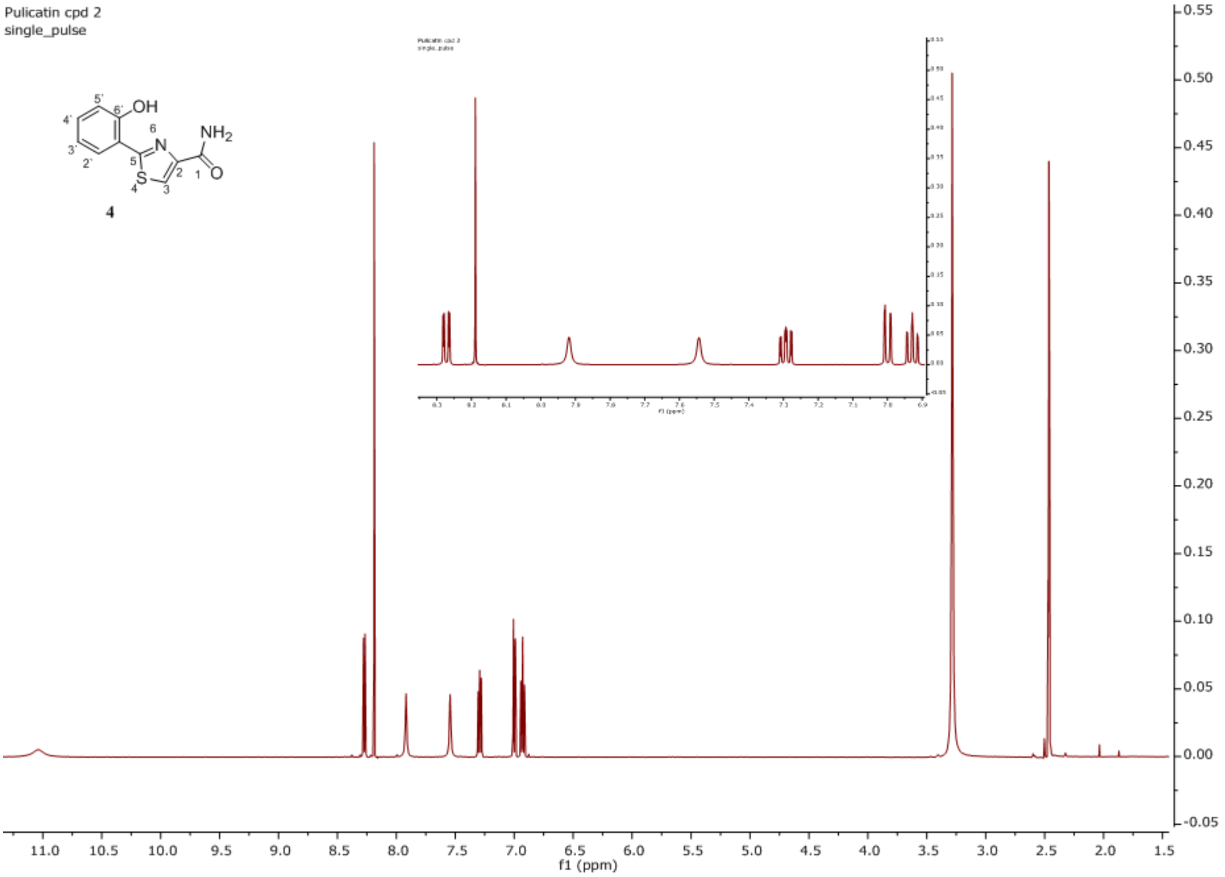

Figure S19. ^{1}H NMR spectrum of compound **4.**

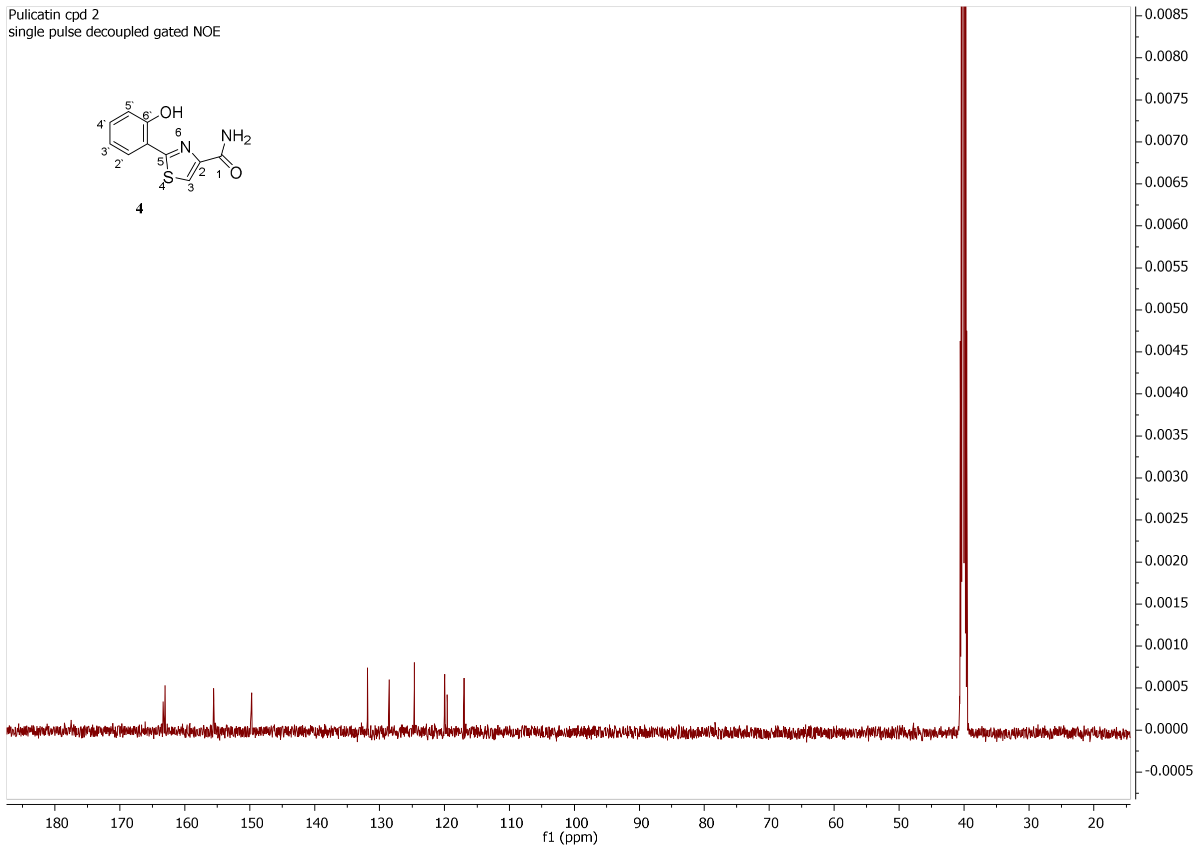

Figure S20. ^{13}C NMR spectrum of compound **4.**

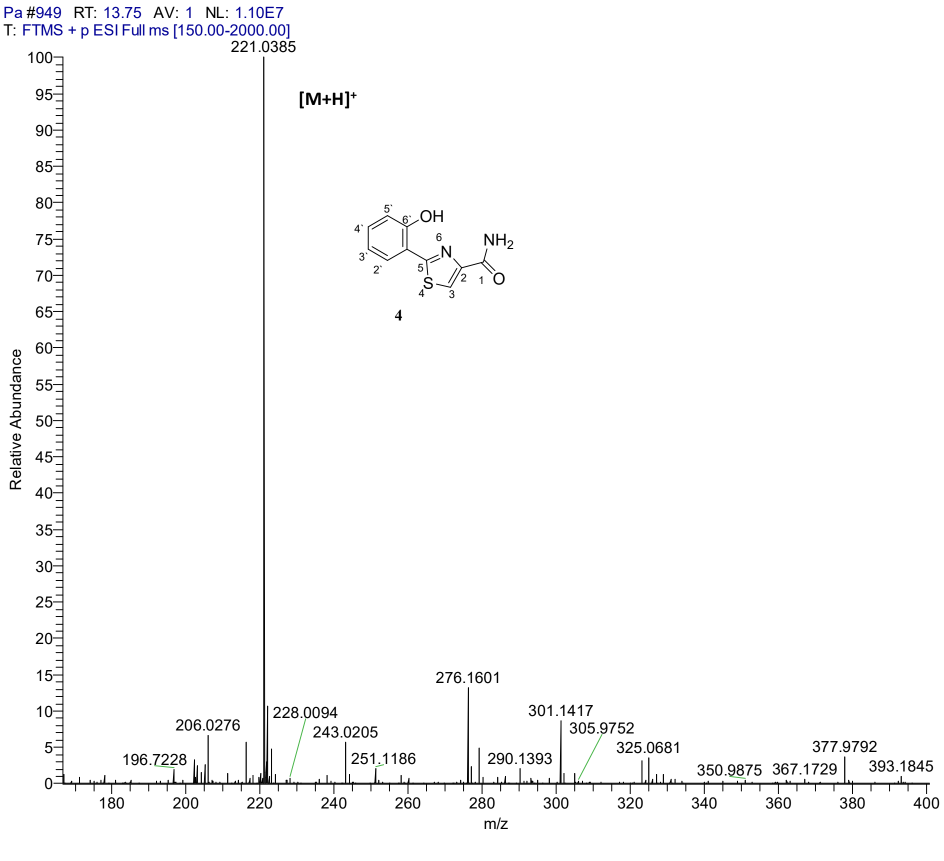

Figure S21. HRMS of compound **4.**
